# The effect of root hairs on exudate composition: a comparative non-targeted metabolomics approach

**DOI:** 10.1007/s00216-022-04475-9

**Published:** 2022-12-22

**Authors:** Martin Lohse, Michael Santangeli, Teresa Steininger-Mairinger, Eva Oburger, Thorsten Reemtsma, Oliver J. Lechtenfeld, Stephan Hann

**Affiliations:** 1 Department of Analytical Chemistry, Helmholtz Centre for Environmental Research – UFZ, 04318 Leipzig, Germany; 2 Department of Forest and Soil Sciences, Institute of Soil Research, University of Natural Resources and Life Sciences, Vienna (BOKU), 3430 Tulln an Der Donau, Austria; 3 Department of Chemistry, Institute of Analytical Chemistry, University of Natural Resources and Life Sciences, Vienna (BOKU), 1190 Vienna, Austria; 4 Institute of Analytical Chemistry, University of Leipzig, 04103 Leipzig, Germany; 5 ProVIS, Centre for Chemical Microscopy, Helmholtz Centre for Environmental Research, UFZ, 04318 Leipzig, Germany

**Keywords:** *Zea Mays* L., Root exudation, Metabolites, Carbon flux, FT-ICR-MS, LC-TOF-MS

## Abstract

Root exudation is a major pathway of organic carbon input into soils. It affects soil physical properties, element solubility as well as speciation, and impacts the microbial community in the rhizosphere. Root exudates contain a large number of primary and secondary plant metabolites, and the amount and composition are highly variable depending on plant species and developmental stage. Detailed information about exudate composition will allow for a better understanding of exudate-driven rhizosphere processes and their feedback loops. Although non-targeted metabolomics by high-resolution mass spectrometry is an established tool to characterize root exudate composition, the extent and depth of the information obtained depends strongly on the analytical approach applied. Here, two genotypes of *Zea mays* L., differing in root hair development, were used to compare six mass spectrometric approaches for the analysis of root exudates. Reversed-phase liquid chromatography and hydrophilic interaction liquid chromatography combined with time-of-flight mass spectrometry (LC-TOF-MS), as well as direct infusion Fourier-transform ion cyclotron resonance mass spectrometry (DI-FT-ICR-MS), were applied with positive and negative ionization mode. By using the same statistical workflow, the six approaches resulted in different numbers of detected molecular features, ranging from 176 to 889, with a fraction of 48 to 69% of significant features (fold change between the two genotypes of > 2 and *p*-value < 0.05). All approaches revealed the same trend between genotypes, namely up-regulation of most metabolites in the root hair defective mutant (*rth3*). These results were in agreement with the higher total carbon and nitrogen exudation rate of the *rth3-*mutant as compared to the corresponding wild-type maize (WT). However, only a small fraction of features were commonly found across the different analytical approaches (20–79 features, 13–31% of the *rth3*-mutant up-regulated molecular formulas), highlighting the need for different mass spectrometric approaches to obtain a more comprehensive view into the composition of root exudates. In summary, 111 *rth3*-mutant up-regulated compounds (92 different molecular formulas) were detected with at least two different analytical approaches, while no WT up-regulated compound was found by both, LC-TOF-MS and DI-FT-ICR-MS. *Zea mays* L. exudate features obtained with multiple analytical approaches in our study were matched against the metabolome database of *Zea mays* L. (KEGG) and revealed 49 putative metabolites based on their molecular formula.

**Graphical Abstract:**

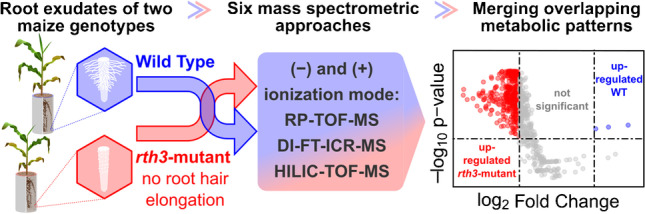

**Supplementary Information:**

The online version contains supplementary material available at 10.1007/s00216-022-04475-9.

## Introduction

Root exudates comprise a mixture of plant-derived primary and secondary metabolites released by roots into the soil [1, 2]. They play a pivotal role in the formation and shaping of the rhizosphere (i.e., the volume of soil affected by living roots). Root exudates drive a range of rhizosphere processes, including nutrient mobilization, soil aggregation, metal detoxification, and stimulation of microbial growth and activity, affecting soil carbon and nutrient cycling. Exudates also promote interactions between plants as well as between plants and other rhizosphere organisms such as bacteria, fungi, protists, or nematodes [3–7].

Organic molecules present in root exudates are often divided into low molecular weight (LMW; < 1 000 Da) and high molecular weight compounds (HMW; > 1 000 Da) [1]. Compound classes such as amino acids, phenolic compounds, sugars and their derivatives, organic acids, and a wide diversity of other secondary metabolites such as flavonoids [8] belong to the LMW compounds, whilst proteins and polysaccharides (i.e., mucilage [9, 10]) account for HMW class [4]. The contribution of each compound class to the total amount of carbon released by the roots varies drastically depending on the plant species or genotype, physiological state, stress conditions, and physical/chemical characteristics of the soil [3, 11–14]. Being directly linked to plant metabolism, metabolomics is the tool of choice to analyze particularly the LMW fraction in root exudates in order to study how root exudation is influenced by genetic and environmental factors [13].

To date, many efforts have been made to identify and characterize individual compounds or compound classes exuded by living and intact roots such as organic acids, monosaccharides, or phenolic acids [15–19]. Non-targeted metabolomics approaches aim at a simultaneous, comprehensive analysis of small molecules of a wide physicochemical range in biological or biogeochemical systems [20]. The compound identity confirmation relies on tentative identification by tandem mass spectrometry coupled with mass spectral libraries research [21–23] or *in silico* approaches [24, 25]. However, absolute quantification of detected compounds remains challenging. Different analytical techniques, such as mass spectrometry (MS) [13, 26, 27], mass spectrometric imaging [28, 29], infrared spectroscopy, and nuclear magnetic resonance (NMR) spectroscopy, have been used for root exudate analysis [2, 16]. To reduce the complexity of the mass spectra and provide a higher selectivity and additional chemical information of the compounds, HRMS-based metabolomics is often coupled with front-end separation techniques (e.g., LC, GC, or capillary electrophoresis (CE)). Due to their ease of use and wide range of accessible polarity, reversed-phase liquid chromatography (RPLC) and hydrophilic interaction liquid chromatography (HILIC) are preferably applied in LC-MS based metabolomics [30–32]. Modern high-resolution mass analyzers offer a variety of operating principles and thus different mass resolving power and mass accuracy. Time-of-flight (resolving power 10,000–60,000; mass accuracy < 5 ppm) and Orbitrap mass analyzers (resolving power 7500–1,000,000, mass accuracy < 1 ppm) [33, 34] have been routinely used for the analysis of root exudates [13, 35–37], while Fourier-transform ion cyclotron resonance mass spectrometry (FT-ICR-MS, resolving power of > 1,000,000, mass accuracy < 1 ppm) is less often reported for this application [38]. Previous studies applying FT-ICR-MS focused on the complex organic matter dynamics such as the effect of model exudates on the microbial transformation of mineral-associated organic matter [39] and the effect of root-derived carbon input on the composition of soil solution organic matter [40]. Gattullo et al. used FT-ICR-MS to identify bisphenol A metabolites in exudates of ryegrass [41], Miao et al. presented a comparison of the molecular components of root exudates of a variety of plant species [38], and Huang et al. investigated the effect of halogenated chemicals on *Zea mays* L. exudates [42].

Due to the large range of physicochemical properties and concentrations expected for different exuded compounds, a combination of different analytical techniques and ionization modes is necessary to give a more comprehensive picture of the metabolic profile [16, 43–45]. The comparison of multiple mass spectrometric platforms has been conducted for metabolites in *Caenorhabditis elegans* [46], lipids in algal secretions [47], and plant response to mechanical wounding [48]. Comparable detection performances between platforms were observed [48], although reporting differences among compounds. This compound-specific difference could lead to a bias when performing differential analysis, which is particularly problematic for non-targeted metabolomics studies. A comparison of non-targeted approaches for plants or root exudates is, to the best of our knowledge, not yet available.

The objective of this study was to compare the composition of root exudates of two soil-grown *Zea mays* genotypes, a wild type (WT) and the *rth3*-mutant. The *rth3*-mutant shows normal root hair initiation, but disturbed elongation compared to the WT. While it is known that hairs increase the carbon input into the soil [49], support rhizosheath formation [50], and are beneficial for the uptake and transport of nutrients [51], a non-targeted metabolomics analysis to investigate the contribution of root hairs to the abovementioned rhizosphere processes has not yet been published.

Furthermore, we aimed to establish if the additional molecular information generated by using a combination of different analytical approaches justifies the additional effort of conducting more measurements and combining the analytical data.

Analytical methods included in this study are direct infusion (DI)-FT-ICR-MS, HILIC-TOF-MS, RPLC-TOF-MS, all using positive (+) and negative (−) electrospray ionization (ESI). The selection of analytical approaches applied in this study mirrors commonly used techniques in the field of metabolomics: direct infusion with an ultra-high resolution instrument (DI-FT-ICR-MS) as well as chromatographic separation hyphenated to a high-resolution analyzer (LC-TOF-MS). Other combinations such as DI-TOF-MS and LC-FT-ICR-MS are applied less often, and we excluded those from our comparison.

The same statistical workflow was applied to the six individual datasets and the results merged for a comprehensive comparison afterwards. By comparing the results of different analytical approaches, we evaluate method-depending differences regarding detected compounds and regulation of matched features after applying the same statistical workflow to the raw data. Overall, this approach will contribute to a higher metabolite coverage and provide a more comprehensive picture of maize root exuded compounds. We expected to observe the same genotype-dependent exudation trend (up-regulation in either WT or *rth3*-mutant) regardless of the analytical technique used for the analysis, which will foster the confidence in non-targeted metabolomics approaches for rhizosphere research—irrespective of the analytical method applied. Overall, this study will help to gain confidence in and robustness of non-targeted data for root exudates as crucial step to extract targets for further structural identification and biochemical interpretation.

## Material and methods

Experimental details on the plant growth conditions, the analysis of chemical parameters of the exudate samples, and the data processing for LC-TOF-MS and DI-FT-ICR-MS can be found in the Supporting Information (SI) to this article.

### Terminology

The term *molecular feature* describes relevant signals consisting of accurate mass and retention time which were obtained by LC-TOF-MS after peak picking, grouping, and feature alignment. A minimum of ≥ 2 matching ions (e.g., isotopologues, adducts) was set as a prerequisite for a molecular feature. Assignment of molecular formulas based on TOF-data is achievable, however, compared to FT-ICR-MS, with less confidence and was hence omitted. *Molecular formulas* were generated from the FT-ICR-MS derived mass to charge (*m/z*) ratios after transformation into molecular masses. If the mass of a molecular feature matched with the mass of a molecular formula (within mass accuracy limits, see SI), the term *compound* was used. A molecular formula can represent multiple chemical structures; however, structural identification of the detected compounds was not in the scope of the present study. This approach corresponds to identification level 4 of the Schymanski scale [23], and in the case of a match of the molecular formula of a *compound* with the KEGG-Database for *Zea mays* L., it was reported as *metabolite*.

### Chemicals

All solvents and reagents used for mobile phase preparation such as methanol, formic acid, acetonitrile (ACN), and ammonium formate were LC‐MS grade and purchased from Sigma-Aldrich (Vienna, Austria). Before measurement, eluents were prepared in LC‐MS grade water containing suitable additives (0.1% *v/v* formic acid; 10 mol L^−1^ ammonium formate, pH adjusted to 3.0 with formic acid). HPLC-grade water (MQW) used for the HPLC-TOF-MS was prepared using Milli-Q® IQ 7000 Ultrapure Lab Water System (Merck, Darmstadt, Germany) combined with a Milli-Q LC-Pak® Polisher. The dilution of the exudate samples for DI-FT-ICR-MS was done with methanol purchased from Biosolve, Valkenswaard, the Netherlands, and HPLC-grade water (MQW) generated from a MilliQ Integral 5 system (Merck, Darmstadt, Germany). The organic matter reference sample Suwannee River Fulvic Acid (SRFA II, International Humic Substances Society) was used for quality control during DI-FT-ICR-MS measurement. Details on the chemicals used to prepare soil column experiments can be found in the Supporting Information to Lohse et al. [40].

### Sampling of root exudates

Root exudates were sampled on the 22nd day of plant growth, 1 h after onset of light, using a soil-hydroponic hybrid approach that combines plant growth in soil with a short hydroponic exudate sampling period [1]. Briefly, six biological replicates of each, the *Zea mays* L. root hair defective mutant (*rth3*), and the corresponding WT [52] intact maize plants with rooted soil columns were gently taken out of their column and carefully rinsed with tap water to remove the soil attached to the roots. Soil-free roots were soaked 3 times in fresh deionized water for 5 min. After that, root exudates were collected for 2 h in 500 mL MQW containing 0.01 g L^−1^ Micropur classic (Katadyn®, Switzerland) as a microbial inhibitor to prevent microbial decomposition of metabolites exuded during the sampling procedure [53].

During the exudates sampling period, plants were placed back into the growth chamber and kept under the same conditions as during the growth period. Subsequently, the sampling solution was filtered (0.45 µm, cellulose acetate, Labsolute), separated into aliquots, frozen, and stored at − 80 °C until mass spectrometric analysis.

All glassware, samplings buckets, and plastic vials were soaked for 20 min in deionized water and then rinsed three times with HQ-water to reduce blank contamination, e.g., from plastic leaching prior use.

The exudate sampling solutions were slightly acidic (pH ~ 6) and contained only a low concentration of dissolved chloride and sulfate (Table S2) and no quantifiable amount of nitrate. This low concentration of inorganic anions confirmed a successful root cleaning protocol.

### Sample preparation and mass spectrometric analysis

#### LC-TOF-MS

Exudate sample aliquots (40 mL) were freeze-dried (− 45 °C; 0.070 mbar, Alpha 1–4 LSCplus, Christ, Osterode am Harz, Germany), reconstituted in 1.2 mL MQW and filtered with 0.2 µm filters (4 mm; cellulose acetate, Nalgene™, USA).

**Fig. 1:**
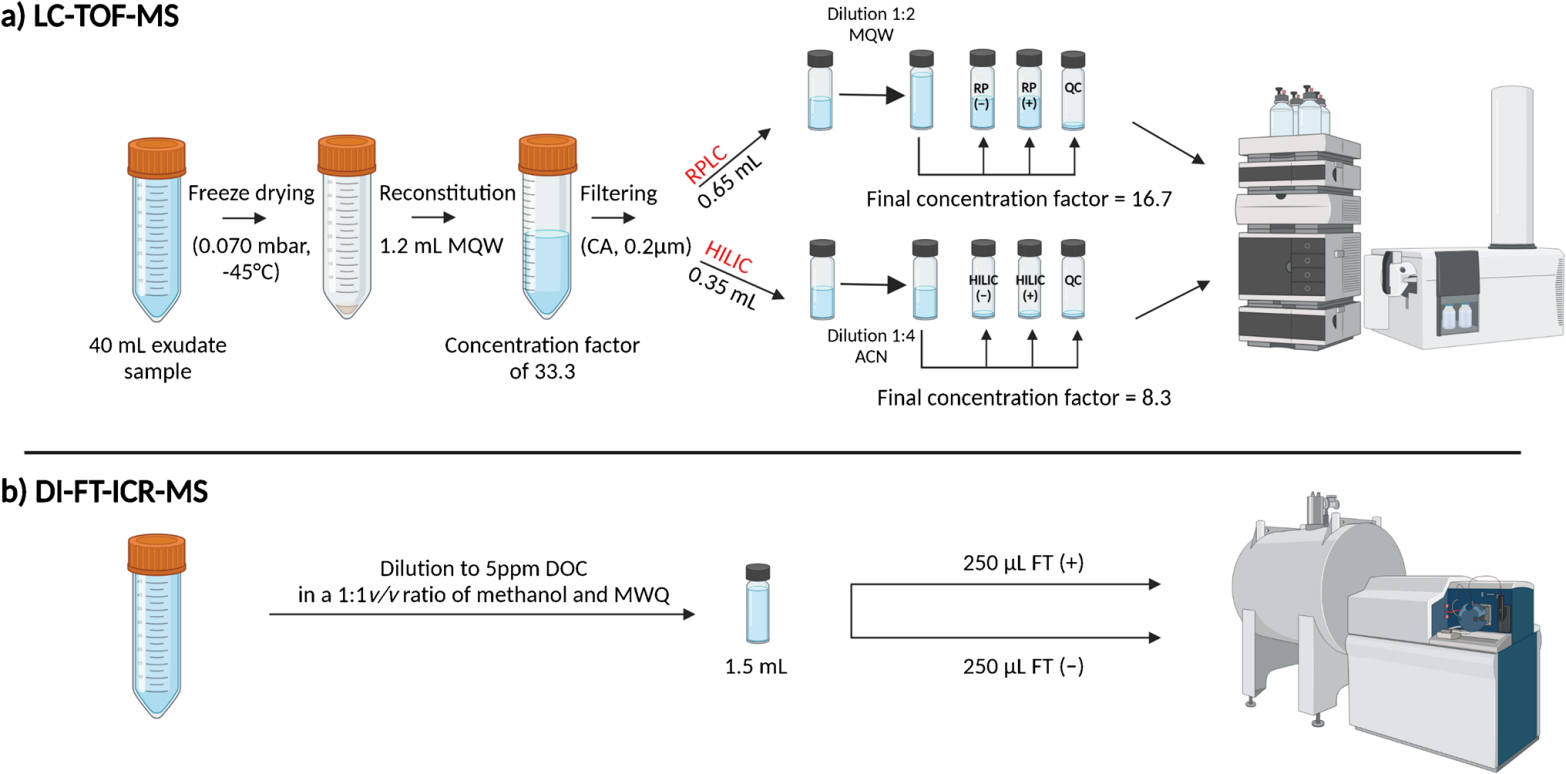
Scheme of LC-TOF-MS root exudate sample preparation. **a** For LC-TOF-MS: the original exudate samples were freeze-dried, reconstituted in MQW, and successively filtered. An aliquot of the resuspended sample (650 µL) was further diluted with MQW and split in RPLC-TOF-MS aliquots. 40 µL from each sample was pooled in the QC sample. For HILIC-LC aliquots, 350 µL of the suspended sample were split into 50 µL aliquots and then further diluted with ACN right before the analysis. HILIC-TOF-MS pooled QCs were prepared by combining 40 µL from each sample, after ACN dilution. An injection volume of 5 µL was used for both LC systems. **b** For DI-FT-ICR-MS: the original exudate samples were diluted to 5 mg C L^−1^ and 250 µL injected at an infusion rate of 10 µl min^−1^ for both polarities. A pooled sample for DI-FT-ICR-MS quality control was prepared using the mean of the dilution factor of all the samples. The figure was created with BioRender.com

Two different chromatographic separation methods were used for the LC-TOF–MS analyzes (RPLC-TOF-MS and HILIC-TOF-MS). A scheme of the sample preparation steps can be found in Fig. 1. For the RPLC-TOF-MS method, reconstituted samples were diluted 1:2 with MQW, while for the HILIC-TOF-MS method, the reconstituted samples were diluted 1:4 with ACN immediately before the measurement. Pooled quality control (QC) samples were prepared by combining equal volumes (40 µL) of each sample. Samples were prepared immediately before the measurement and stored at 4 °C during the entire measurement sequence. Samples were injected in randomized order. QCs were measured after every four samples. These QCs were used for monitoring the performance of the LC-TOF-MS system. A UHPLC system with binary pump (Infinity II, Agilent Technologies, Santa Clara, USA) and an HTC-PAL autosampler unit with cooled sample trays (CTC analytics AG, Zwingen, Switzerland) was employed. For the separation of metabolites, Atlantis T3 C18 and XBridge BEH amide® column (both 2.1 × 150 mm, 3 µm particle size for Atlantis T3 and 3.5 µm for XBridge amide, Waters Corporation, Milford, USA), were used for RPLC and HILIC, respectively.

An additional pump (1260 series, Agilent Technologies, Santa Clara, CA) was used to provide a reference mass solution for online mass calibration (*m/z* = 121.0509 and 922.0098 (+); *m/z* = 112.9856 and 1033.9881 (−)) (ESI-L tuning mix, Agilent Technologies, Santa Clara, CA) and was connected to the secondary sprayer of the ESI interface (Dual Jet Stream®, Agilent Technologies, Santa Clara, CA) of the TOF-MS (6230B, Agilent Technologies). The LC-TOF–MS system was controlled by MassHunter acquisition software (version 10.1, Agilent Technologies, Santa Clara, CA).

The RPLC-TOF-MS method was adapted from [54]. Operational conditions for the chromatography system and TOF–MS settings are listed in Table S1. The mobile phases used for the RPLC-TOF-MS separation were (A) MQW with 0.1% v/v formic acid and (B) 100% methanol. Initial gradient conditions (100% A) were kept for 2 min and then ramped to 40% B within 8 min and kept for 2 min before starting the cleaning step, where the mobile phase composition was increased to 100% B within 0.1 min and kept for 2 min. The re-equilibration phase consisted of a decrease of B from 100 to 0% within 0.1 min, followed by 5.9 min in 0% B to restore the initial conditions. The total analysis time was 20 min per injection, with a constant flow rate of 200 μL min^−1^ and 40 °C column oven temperature. A full loop injection was applied using a 5 μL stainless steel injection loop with a loading volume of 15 μL.

The mobile phases used for the HILIC-TOF-MS separation were (A) 10 mmol L^−1^ ammonium formate, adjusted to pH 3.0 with formic acid, and (B) 99.9% v/v ACN with 0.1% v/v formic acid. Initial gradient conditions (90% B) were kept for 2 min, then decreased to 35% B within 10 min, and finally returned to starting conditions over 0.3 min followed by a re-equilibration phase of 5.7 min. The total analysis time was 18 min per injection, with a constant flow rate of 200 μL min^−1^. An injection volume of 5 μL and a column temperature of 40 °C were used. An inline VHP 0.2 μm steel filter (IDEX, Northbrook, USA) was used for both the RPLC-TOF-MS and HILIC-TOF-MS column.

Instrument tuning and mass calibration were performed according to the manufacturer’s instructions. For TOF–MS detection, mass range setting *m/z* 90 − 1700 were recorded in positive and negative polarity. The 2 GHz extended dynamic range mode with a spectral acquisition rate of 2 Hz was used for all measurements. Mass resolving power was ≥ 8000 for *m/z* 322 (+) and *m/z* 301 (−).

#### DI-FT-ICR-MS

For DI-FT-ICR-MS analysis, exudate samples were diluted to 5 mg C L^−1^ in a 1:1 (*v/v*) mix of methanol and MQW without any additional sample preparation steps (Fig. 1).

The added silver salt from the microbial inhibitor did not result in detectable silver adducts in the mass spectrum. Blanks and pooled samples for quality control were prepared in the same way using the mean of the dilution factor of all the samples (Table S2).

An FT-ICR mass spectrometer equipped with a dynamically harmonized analyzer cell (solariX XR, Bruker Daltonics Inc., Billerica, MA, USA) and a 12 T refrigerated actively shielded superconducting magnet (Bruker Biospin, Wissembourg, France) was used. The mass spectrometer was controlled with ftmsControl 2.2.0 (Bruker Daltonics, MA). Mass spectra were recorded in the mass range setting *m/z* 74 − 1 000 in magnitude mode (eight megaword time domain). External mass calibration was performed with SRFA. A standard ESI source (Apollo II, Bruker Daltonics, Billerica, MA) was used for the direct infusion measurement via an autosampler (injection volume: 250 µL, flow rate: 10 µL min^−1^). The capillary voltage was set to 4.3 kV for negative mode measurements and 5.0 kV in positive ionization mode, respectively. Parameters for the ESI source were as follows: dry gas temperature: 200 °C, dry gas flow rate: 3.0 L min^−1^, nebulizer gas flow rate: 1.0 bar. For one mass spectrum, 512 scans were co-added with an ion accumulation time of 30 ms. Due to the ultra-high mass resolving power of the FT-ICR-MS (> 1,000,000 at *m/z* 400), the unambiguous assignment of molecular formulas is possible (see SI for information regarding the data processing).

### Statistical analysis

For normalization of the acquired mass spectrometry data, the absolute mass peak intensity for each molecular feature or molecular formula was divided by the root dry weight (dwt) of the respective plant to compensate for different amount of biomass, and thus exuded C amount, between genotypes and replicates (Fig. S1). This adjusted intensity was further multiplied by the respective dilution factor prior measurement: For the LC-TOF-MS methods, it was always 1, whereas for the DI-FT-ICR-MS, the dilution factor varied between 2.4 and 5.7, depending on the DOC concentration (Table S2) that was applied. For blank samples and quality control samples, the mean of the dwt of all the plants was used (Table S3). 

A list of samples and a detailed workflow of the applied statistical differential analysis can be found in Table S4 and Fig. S1, respectively. The exudate sample from the plant in soil column 3 was excluded from all further evaluations due to significantly different biomass as compared to the other replicates (Table S3).

Further data filtering steps were applied (Fig. S1):

- Blank correction: Molecular features/formulas were considered, if the mean of the normalized peak intensity across all the samples was at least ten times higher than the mean of the blanks.
- Frequency filter: Molecular features/formulas were included if present in ≥ *n* − *1* biological replicates for each genotype.
- RSD filter: Molecular features/formulas were included if the relative standard deviation (RSD) of their normalized peak intensity was < 35% in at least one of the two genotypes.

Differences in the carbon and nitrogen exudation rate between the WT and *rth3* genotype were evaluated by an unpaired *t* test using GraphPad Prism 8.0.2 (GraphPad Sofware, San Diego, CA, USA). To select significant molecular features/formulas, an unequal variances *t* test (Welch’s *t* test with Benjamini-Hochberg false discovery rate correction [55]) was conducted on log-transformed, dwt-scaled intensities to compare the two genotypes. Significance level *α* was set to 0.05. Statistical analysis of LC-TOF-MS data was performed in GraphPad Prism 8.0.2 (GraphPad Sofware, San Diego, CA, USA). Statistical analysis of molecular formulas data for DI-FT-ICR-MS analysis was carried out using R (Version 4.0.3) [56]. Aggregated molecular descriptors were calculated on the number-based mean. Finally, molecular features/formulas, with a minimum fold change of 2 between genotypes, and a *p*-value < 0.05 were considered as significant compounds.

The described data processing and statistical workflow was independently applied to all platforms. As such it can be reproduced, even if one or the other approach is not available. The workflow provides method/platform-dependent assessment of metabolite patterns of complex biological systems considering biological replication, quality control, and statistical analysis/hypothesis testing.

### Data merging for different MS approaches

In order to reveal differences in metabolite coverage/identification that go beyond this level of confidence in analytical results, e.g., due to the utilization of different analytical approaches (e.g., DI- vs LC-), we conducted further data processing steps.

Molecular features from LC-TOF-MS were merged with the molecular formulas from DI-FT-ICR-MS analysis based on molecular mass [57] and a formula assignment was conducted for the TOF-MS data (identification confidence level 4 [23]).

This allowed comparing significant molecular features/formulas of all six analytical approaches (RPLC-TOF-MS (+)/(−), HILIC-TOF-MS (+)/(−), and DI-ICR-MS (+)/(−)). Due to the overall higher mass accuracy (resulting in smaller ppm thresholds), all the significant molecular formulas of the DI-FT-ICR-MS (+)/(−) measurements were analyzed separately. The resulting matched dataset was filtered to remove duplicates (matching the same molecular formula with itself and permutations) and further filtered based on the molecular mass difference. When the mass difference was below 5 ppm (estimated maximum error of TOF-MS) for matches of LC-TOF-MS and DI-FT-ICR-MS and 0.5 ppm for matches between DI-FT-ICR-MS (+) and DI-FT-ICR-MS (−), the match was further processed. These filter criteria lead exclusively to unique matches (i.e., one molecular feature of an LC mode matches just one molecular formula of the FT-ICR-MS analysis). Matches were only further analyzed when both analytical platforms, namely TOF-MS and FT-ICR-MS, indicated up-regulation in the *rth3*-mutant for the same mass.

The *rth3*-mutant up-regulated and matched features/formulas from the first comparison were used to include the retention time information. Compounds showing the same RT (ΔRT < 0.1 min) and mass (< 5 ppm) within the same chromatographic method but with different ionization polarity were considered the same compound, and the mean of the two RT was calculated. Compounds detected by different chromatographic methods having the same mass, but different RT were always considered different compounds. For merging of the results from different analytical approaches, R (Version 4.0.3) was used.

For generating a list of relevant Kyoto Encyclopedia of Genes and Genomes (KEGG) IDs, the KEGG-compound database was used to match the compounds based on their molecular formula. Detected and assigned sodium adducts were represented as the protonated molecule and possible duplicates were removed for the database matching. Via the KEGG (pathway) Mapper – Search [58], putative metabolites of *Zea mays* L*.* (organism code: zma) were assigned to the KEGG IDs. Since molecular formulas were compared, tentative structures for possible metabolites can be proposed [23].

## Results and discussion

### Plant parameters and total carbon and nitrogen exudation rates

Total carbon and nitrogen content of the exudate samples and plant morphological parameters were determined to compare differences in root exudation and growth performances between the two *Zea mays* L. genotypes.

**Fig. 2:**
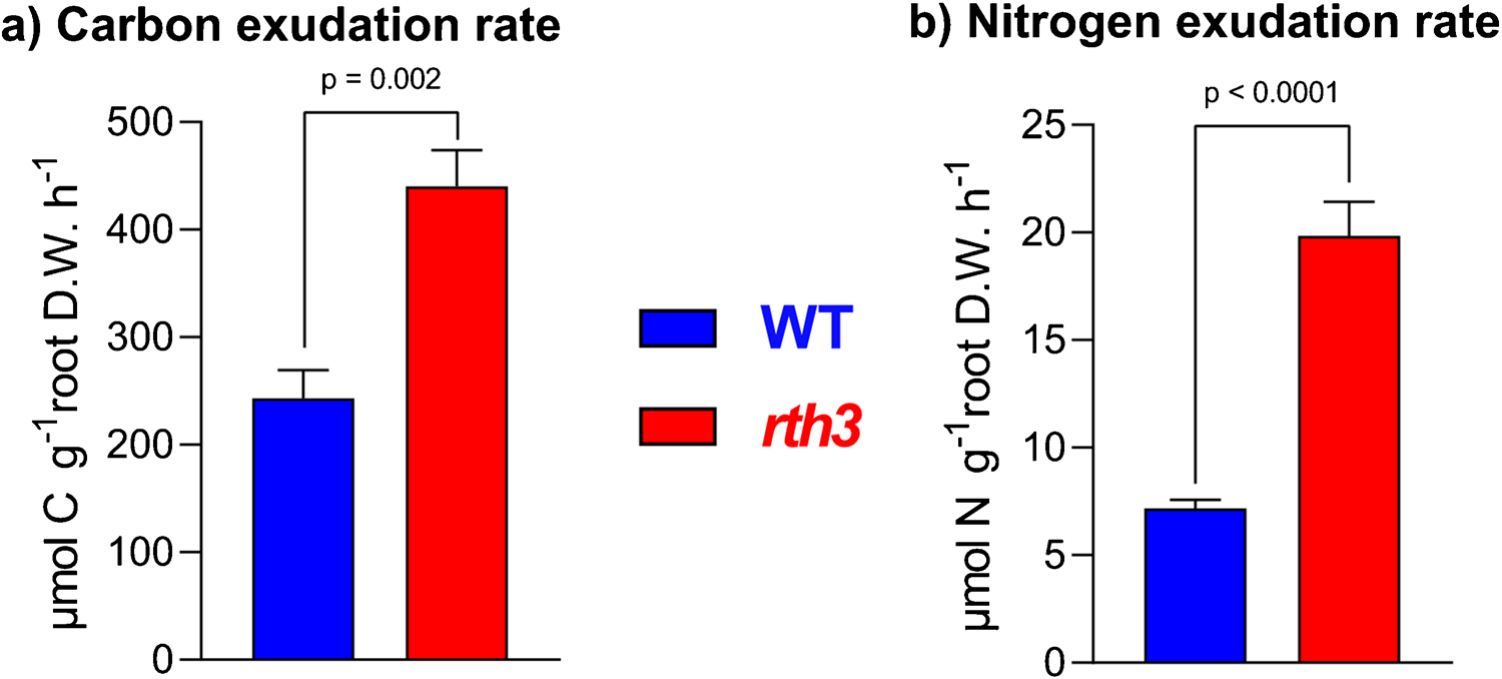
Exudation rates of the WT compared to the *rth3*-mutant for (**a**) carbon and (**b**) nitrogen. Data are expressed as mean ± standard error (WT: *n* = 6, rth3: *n* = 5)

The root hair defective *rth3-*mutant and the corresponding WT showed a significant difference in both shoot and root biomass (Table S3). The total carbon exudation rate was on average 440 µmol carbon g_root dwt_^−1^ h^−1^ for the *rth3*-mutant, while the WT with higher mean biomass released 243 µmol carbon g_root dwt_^−1^ h^−1^. Also, the nitrogen exudation rate differed significantly, with 19.8 and 7.18 µmol nitrogen g_root dwt_^−1^ h^−1^ for the *rth3*-mutant and the WT, respectively (Fig. 2 and Table S2). These results were unexpected, considering that root hairs were proposed to contribute significantly to root exudation [49]. Our results suggest that lack of root hairs and their plant beneficial functions (e.g., increased absorption surface) is compensated by a higher root exudation rate in *rth3,* which could trigger increased microbial colonization and turnover as well as increased nutrient solubility in the rhizosphere of the *rth3*-mutant.

### Non-targeted analysis of root exudates

The metabolites present in exudates of the two maize genotypes (WT and *rth3*) were analyzed with six analytical approaches. A summary of the total number of detected features is shown in Table 1.

**Table 1:** Overview of the total number, significant, and genotype-specific up-regulated features in the exudate samples resulting after filtering and differential statistical analysis. The significant molecular features (fold change > 2 and *p*-value < 0.05) are further separated by genotype-specific regulation and described by the fraction of significant features, as well as with the minimum/maximum/mean molecular mass. The percentage of *rth3*-mutant up-regulated molecular features/formulas is also given. Cf. also Figs. 3 and S2–S4

	DI-FT-ICR-MS (−)	DI-FT-ICR-MS (+)	HILIC-TOF-MS (−)	HILIC-TOF-MS (+)	RPLC-TOF-MS (−)	RPLC-TOF-MS (+)
Total features	602	889	238	176	751	266
Significant features	351	611	121	93	361	169
Molecular mass (Da) min. – max. (mean)	100–698 (318)	119–666 (366)	105–656 (316)	111–569 (313)	112–917 (403)	111–774 (312)
Fraction of significant features (%) of the total molecular features	58%	69%	51%	53%	48%	64%
Up-regulated in WT	95	166	3	1	6	0
Up-regulated in *rth3*-mutant	256	445	118	92	355	169
Fraction of *rth3*-mutant up-regulated features (%) of the significant features	73%	73%	98%	99%	98%	100%

**Fig. 3:**
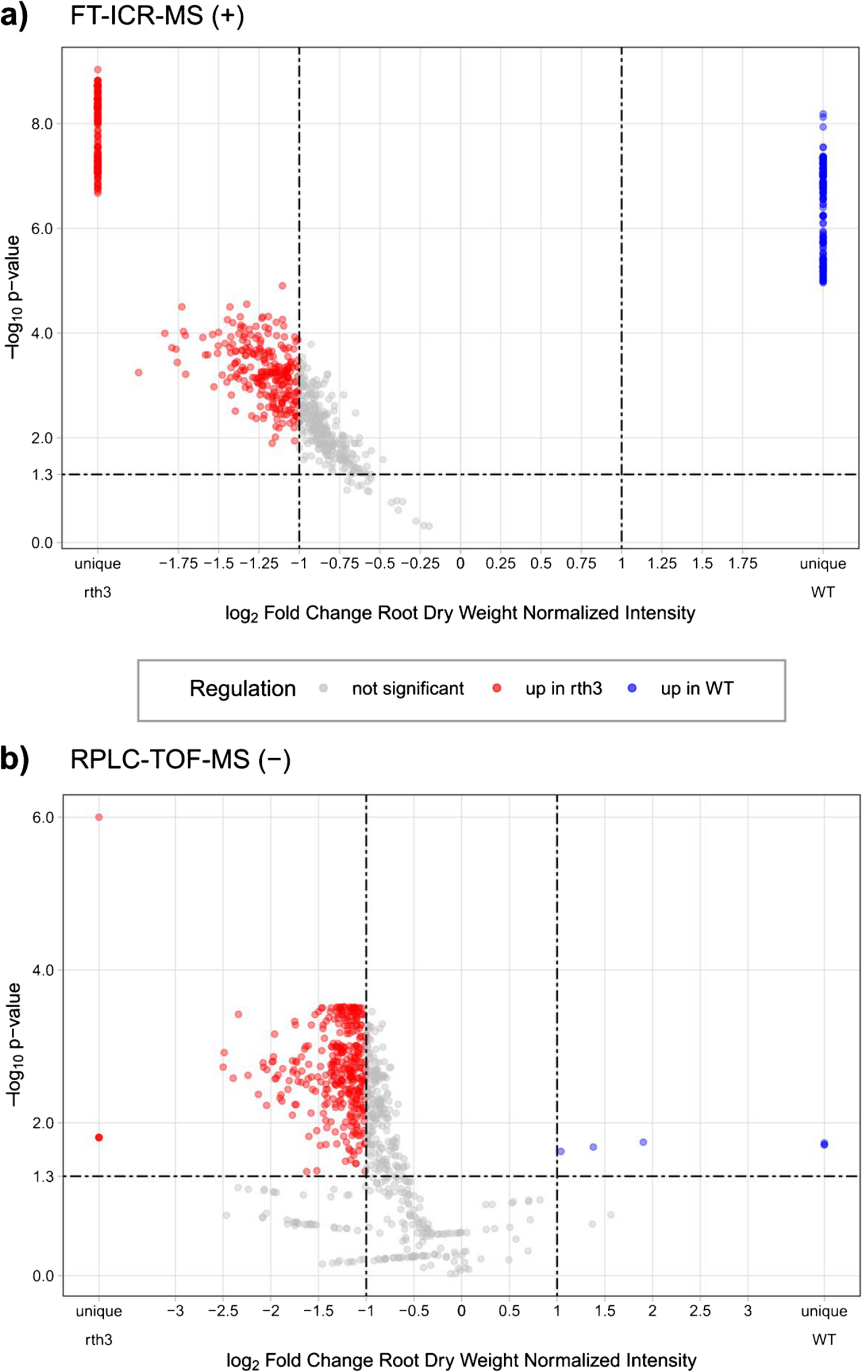
Volcano plots of the comparison of the WT vs *rth3-*mutant for (**a**) FT-ICR-MS (+), based on 889 molecular formulas; (**b**) RPLC-TOF-MS (−) based on 751 molecular features. Molecular formulas/features up-regulated in WT or rth3-mutant that passed both cut-offs (*p*-value < 0.05, FC > 2) are shown as blue and red dots, respectively. Non-significant molecular features are represented as grey dots. The *p*-value threshold (0.05) and FC threshold (2) lines are shown in the graphs as dashed lines. Additional volcano plots visualizing the same trend for all the analytical approaches are shown in the SI (Figs. S2 to S4)

The total number of detected features varied depending on the analytical methods used for the analysis. Nevertheless, most of the detected molecular features/formulas showed up-regulation in the *rth3* plants (73–100%, Table 1), and this trend was the same independently from the analytical method and reflected the higher carbon and nitrogen exudation rate determined in the *rth3*-mutant (Fig. 2).

For LC-TOF-MS, the data showed a highly dominant fraction of *rht3* up-regulated features, irrespectively of the chromatographic method and ionization used.

#### DI-FT-ICR-MS

A percentage of significant molecular formulas was found with DI-FT-ICR-MS (between 58% (−) and 69% (+)), matching well with the results for the LC-TOF-MS results (48–64%) (Table 1). In total, 962 significant molecular formulas (898 different molecular formulas) were identified in both genotypes for two ionization modes (Figs. 3a and S2). Only 64 molecular formulas were detected jointly in positive and negative mode. This small overlap illustrates the need to apply different ionization polarities to cover the molecular diversity of root exudates with DI-FT-ICR-MS.

**Fig. 4:**
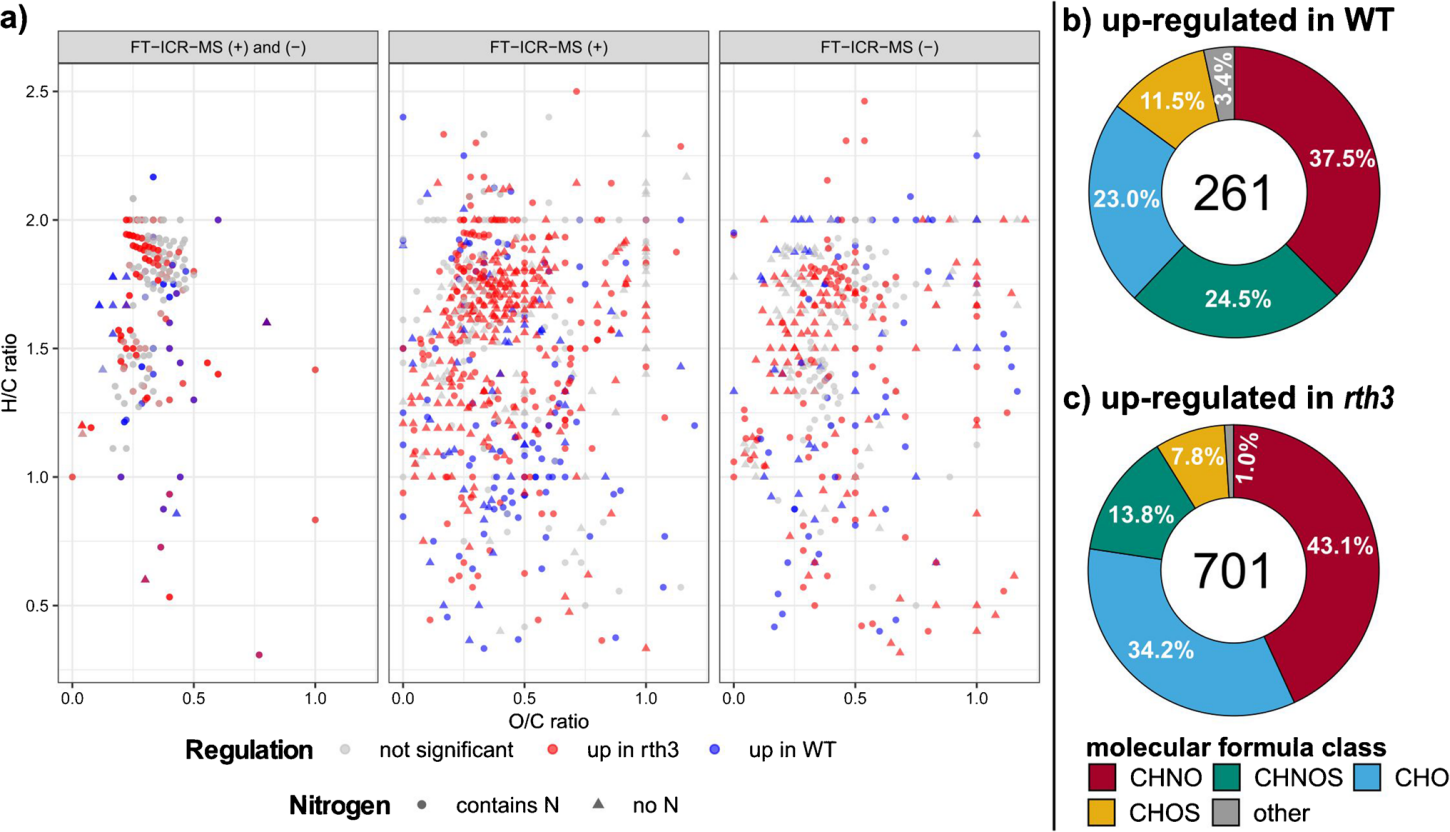
Comparison of molecular formulas assigned for negative and positive ionization mode FT-ICR-MS measurements and molecular formula classes up-regulated in the genotypes. **a** *Van Krevelen* diagram comparing molecular formulas assigned solely for negative mode or positive mode measurements and molecular formulas detected jointly with the two polarities. **b** and **c** Fraction of molecular formula classes for WT and *rth3*-mutant up-regulated molecular formulas (CHNO: red, CHNOS: cyan, CHO: blue, CHOS: yellow, other: gray). The number in the center of the charts is the total number of significant molecular formulas

Despite the small overlap of molecular formulas detected with both DI-FT-ICR-MS (+) and DI-FT-ICR-MS (−), in the van Krevelen diagram (i.e., elemental ratios: H/C and O/C-ratio, Fig. 4a), or the double bond equivalent (DBE) vs mass plot (Fig. S5), the molecular formulas detected with DI-FT-ICR-MS (+) (O/C: 0.41, H/C: 1.49, DBE: 5.50) and DI-FT-ICR-MS (−) (O/C: 0.43, H/C: 1.46, DBE: 5.70) averaged at similar values.

Molecular formulas detected with DI-FT-ICR-MS (−) also averaged at a not significantly higher N/C-ratio (0.15) compared to the molecular formulas solely detected with DI-FT-ICR-MS (+) (0.13). The average molecular mass increased significantly (*p*-value < 0.001) from 318 Da for DI-FT-ICR-MS (−) to 366 Da for DI-FT-ICR-MS (+). The difference in the averaged molecular mass between the two DI-FT-ICR-MS ionization polarities (318 to 366 Da) may be partly related to sodium-containing molecular formulas in the positive mode. In conclusion, DI-FT-ICR-MS (−) allows the detection of smaller molecules with higher ratio of heteroatoms (O, N) and higher DBE. The general trends for the O/C-ratio, molecular mass, and DBE for the two ionization modes were previously described by Miao et al. for maize exudates using FT-ICR-MS despite the different data treatment strategy compared to this study [38].

Using the number-based averaged molecular descriptor for the two genotypes indicates that the WT releases fewer up-regulated compounds (*n* = 261) which are significantly smaller (314 Da, *p*-value < 0.001), more oxygen-rich (O/C-ratio: 0.44, *p*-value > 0.05), and significantly more unsaturated (H/C ratio: 1.35 (*p*-value < 0.001), DBE: 5.98 (*p*-value < 0.05)) compared to the *rth3*-mutant (molecular mass: 361 Da, O/C: 0.41, H/C: 1.53, DBE: 5.42, *n* = 701).

The fraction of sulfur- and nitrogen-containing formulas are significantly higher (*p*-value < 0.05) for the WT up-regulated (*N*: 169 (65%) of total 261 molecular formulas, N/C-ratio: 0.15, Fig. 4b) compared to the *rth3*-mutant up-regulated (*N*: 405 (58%) out of 701 molecular formulas, N/C-ratio: 0.13, Fig. 4c). The higher absolute number of *rth3*-mutant up-regulated molecular formulas containing nitrogen corresponds well with the higher nitrogen exudation rate observed for this genotype (Fig. 2 and Fig. S6). The high fraction of nitrogen-containing molecular formulas in root exudates (58–65%) might be the reason for the higher number of molecular masses detected with FT-ICR-MS (+) compared to the negative mode measurement (Fig. S6). Due to the more efficient ionization of molecules with proton-acceptor functional groups (such as amino-groups), a more sensitive detection is possible in positive mode [59], and a higher number of nitrogen-containing species can be detected [60]. An even higher fraction (51%, combination of FT-ICR-MS (+) and (−)) of the CHNO formula class in maize root exudates was observed previously [38] compared to the results in this study (38–43%, Fig. 4b and c).

#### LC-TOF-MS

The HILIC-TOF-MS analyzes results showed fewer detected features than RPLC-TOF-MS, independently from the ionization.

The mass-vs-RT plots (Fig. S7), visualizing both RPLC-TOF–MS and HILIC-TOF-MS chromatographic separation, demonstrate that the detected molecular features cover the entire chromatographic run time representing the physicochemical diversity of root exudates, in particular its hydrophobicity.

The difference in detected compounds between the two LC techniques observed in this study suggests that weakly polar or hydrophobic compounds comprise a large fraction of the maize root exudates, for which RPLC-TOF-MS is more appropriate than HILIC. A higher number of detected features with RPLC-TOF-MS as compared to HILIC-TOF-MS was also reported by Wernisch et al. [61] for a broad range of metabolites.

RPLC-TOF-MS (−) showed the highest number of detected (751) as well as statistically significant molecular features (361, Figs. 3b and S4). The percentage of significant molecular features over the total detected was comparable among the four LC modes, with RPLC-TOF-MS (+) showing the highest percentage (64%) (Table 1). Interestingly, higher numbers of detected and significant features were found in both RPLC-TOF-MS and HILIC-TOF-MS in (−) compared to (+) ionization, suggesting that a substantial fraction of the exudate samples detected with the LC methods consisted of compounds with acidic functional groups such as organic acids and phenols showing a higher ionization yield for (−) ionization mode [62]. Interestingly, the rth3-mutant up-regulated features detected with HILIC-TOF-MS had a significantly lower average mass (315 Da, *n* = 210, *p*-value < 0.001) compared to RPLC-TOF-MS (373 Da, *n* = 524).

#### Comparison between DI-FT-ICR-MS and LC-TOF-MS

Averaging the molecular masses for the two genotypes independent of the analytical approach revealed a significantly lower mass for the WT (317 Da, *n* = 271, *p*-value < 0.001) compared to the *rth3*-mutant (359 Da, *n* = 1435). A high number of unique molecular formulas (i.e., significant molecular formulas only present in one genotype) was detected for the FT-ICR-MS analysis (DI-FT-ICR-MS (−): 195 and DI-FT-ICR-MS (+): 353). For the LC-approaches, most of the molecular features were detected in both genotypes, and only a few molecular features were unique for one of the two genotypes (molecular features: HILIC-TOF-MS (−): 4, HILIC-TOF-MS (+): 3, RPLC-TOF-MS (−): 8, and RPLC-TOF-MS (+): none).

The apparent differences in absolute numbers, unique features, and fractions of *rht3* up-regulated features between the two MS-platforms (LC-TOF-MS and FT-ICR-MS) (Table 1) might be related to the sample preparation and/or the chromatographic separation used with the TOF-MS but not the DI-FT-ICR-MS. For LC-TOF-MS, root exudates samples were lyophilized, and even though lyophilization is a standard enrichment procedure in metabolomics studies [63–65], it can affect the stability of selected groups of metabolites (e.g., phenolics and organic acids) [65–67], potentially increasing the variability between exudate sample replicates. A higher RSD might lead to an exclusion of features according to the RSD quality filter. Although HILIC and RP are offering some degree of orthogonality, not all compounds present in the sample are retained and amenable to this combination of methods, especially when the sample is a complex mixture of different compounds [68, 69], as in the case of root exudates. Hence, some of the compounds detected with the DI-FT-ICR-MS may have eluted in the void volume of the LC-separations, which was not considered for evaluation of the TOF-MS data. In addition, non-ideal peak shape may occur in particular for HILIC-TOF-MS [61, 70, 71], and these features would be removed during peak integration and picking as well.

For the LC-approaches, more molecular features were detected in (−) ionization mode compared to the (+) mode. Interestingly, this trend contrasts with the FT-ICR-MS results, where more *rth3* up-regulated features were detected in (+) mode. Possible reasons for that difference can be related to differences in the matrix effects or the presence of isobaric compounds which might affect DI-FT-ICR-MS more strongly compared to LC-hyphenated approaches.

Comparing the LC-TOF-MS raw data for the 95 formulas uniquely detected as up-regulated in the WT with FT-ICR-MS (−), we observed that a small fraction of the 95 formulas were also detected with the LC-approaches (16 with RPLC-TOF-MS and 7 with HILIC-TOF-MS) (SI Excel table, Tab: Data_WT_Upregulated). Nevertheless, though detected, all those features were excluded from further evaluation either because they did not pass the quality filters used in the MFE (3 features) and statistical workflow (3 features) or because they showed a different regulation (17 features; i.e., *rth3-*up-regulated instead of WT-up-regulated, SI Excel table, Tab: Data_WT_Upregulated). Moreover, the Molecular Feature Extraction (MFE, see SI) used for the LC-approaches aligns and groups different ion species or dimers in one feature, decreasing the total number of detected features for the LC-approaches. For the DI-FT-ICR-MS such a grouping of features was not applied due to the lack of retention time information and thus unclear identity of the ion species beyond the molecular formula. Based on this comparison, we would conclude that the difference in detected features between analytical approaches was not an artifact of our statistical workflow, and the high variety of detected features (i.e., low degree of shared features) was rather determined by the differences between two analytical approaches.

### Compound commonalities between the MS platforms

#### Overlap between the methodological approaches based on accurate mass measurement

While DI-FT-ICR-MS offers high confidence for molecular mass determination and molecular formula assignment but lacks (retention) time dimension, in LC-TOF-MS, the chromatographic separation before mass spectrometric detection provides additional RT-information despite lower mass accuracy [57, 72].

To find matches between the mass spectrometric approaches, the molecular masses detected with the LC-TOF-MS were compared to those detected with FT-ICR-MS (Table S5). The goal was to find significant compounds jointly detected with different analytical methods. Combining LC-TOF-MS with DI-FT-ICR-MS data can be beneficial to remove method-specific artifacts, validate the feature detection algorithms, and deliver additional structure related information (e.g., via polarity assessment from chromatographic results) to support root exudate characterization.

In total, 254 pairwise matches based on the molecular mass were found, 195 of which showed an up-regulation in the *rth3*-mutant for both approaches (Table S5). Finding the same mass with the same trend in metabolite expression with two independent approaches was used as additional evidence for compound filtering. The highest number (38) of matches was found between FT-ICR-MS (+) and FT-ICR-MS (−). Partly due to the higher number of significant compounds detected with RPLC-TOF-MS, more matches were shared between FT-ICR-MS and RPLC-TOF-MS (108 matches for all combinations) compared to HILIC-TOF-MS (49 matches) (Table S5).

Only 9 shared molecular formulas were found to be up-regulated in the WT, and they were the result of the DI-FT-ICR-MS (+) and DI-FT-ICR-MS ( −) matching, while they were not detected by any LC-TOF-MS method (Table S5). Because there was no overlap with any LC approach (i.e., no RT information), those molecular formulas were neglected during the subsequent data analysis. The remaining 50 from the total of 254 pairwise matches showed a different regulation between approaches (i.e., same mass had an up-regulation in the WT with one approach, while the other approach revealed up-regulation in the *rth3-*mutant), and this was most likely due to the presence of different isobaric compounds showing contrasting regulation between the two genotypes, thus were excluded from further analysis. Only matches where both mass spectrometric approaches showed up-regulation in the *rth3*-mutant were further processed.

Just a small fraction of the *rth3*-mutant up-regulated molecular formulas detected by FT-ICR-MS had an overlap with the features detected with LC-TOF-MS (Table 2). The small fraction of matched features (20–79 features, 13–31%) between two analytical approaches indicates that combinations of more analytical approaches help to further extend the range of detectable compounds. The small overlap cannot be explained by noise in the data due to the strict statistical filtering applied for our metabolomics approach (Fig. S1). However, differences in the sample preparation or applied front-end separation approach (DI vs LC) might explain the low fraction of matches.

**Table 2:** *rth3*-mutant up-regulated compounds after matching based on molecular mass. The total number of *rth3*-mutant up-regulated compounds and fractions of pairwise matches of the analytical approaches HILIC/RPLC/FT-ICR-MS with FT-ICR-MS based on molecular mass. The column *Number of matches* contains duplicates if a compound was matched with > 1 other mode. The number equals the sum of compounds found for each mode as reported in Table S5

Analytical approach	*rth3*-mutant up-regulated compounds	Number of matches	Unique *rth3*-mutant up-regulated compound matches	Fraction match (unique/ total *rth3*-mutant up-regulated)
DI-FT-ICR-MS (−)	256	129	79	31%
DI-FT-ICR-MS (+)	445	104	64	14%
HILIC-TOF-MS (−)	118	22	20	17%
HILIC-TOF-MS (+)	92	27	24	26%
RPLC-TOF-MS (−)	355	55	45	13%
RPLC-TOF-MS (+)	169	53	44	26%

Taken together, the number of compound commonalities between the MS platforms suggests that a combination of different analytical approaches might be helpful for identification. However, to get an impression of exudation trends, only one approach would suffice.

#### Overlap between the methodological approaches based on accurate mass and retention time

To further narrow down relevant and shared compounds, the LC-TOF-MS retention time information was considered and combined with the accurate mass and molecular formula information from FT-ICR-MS. In total, 92 *rth3*-mutant up-regulated molecular formulas from FT-ICR-MS were matched with a mass, in at least one of the LC-TOF-MS analysis (35 for HILIC-TOF-MS, 57 RPLC-TOF-MS). The matching, including retention time as an additional filter, revealed that a total of 111 compounds (i.e., same molecular formula detected at different retention times within the same LC mode) present in the LC-TOF-MS dataset were found to overlap with FT-ICR-MS data. The complete list of compounds can be found in Table S6.

**Fig. 5:**
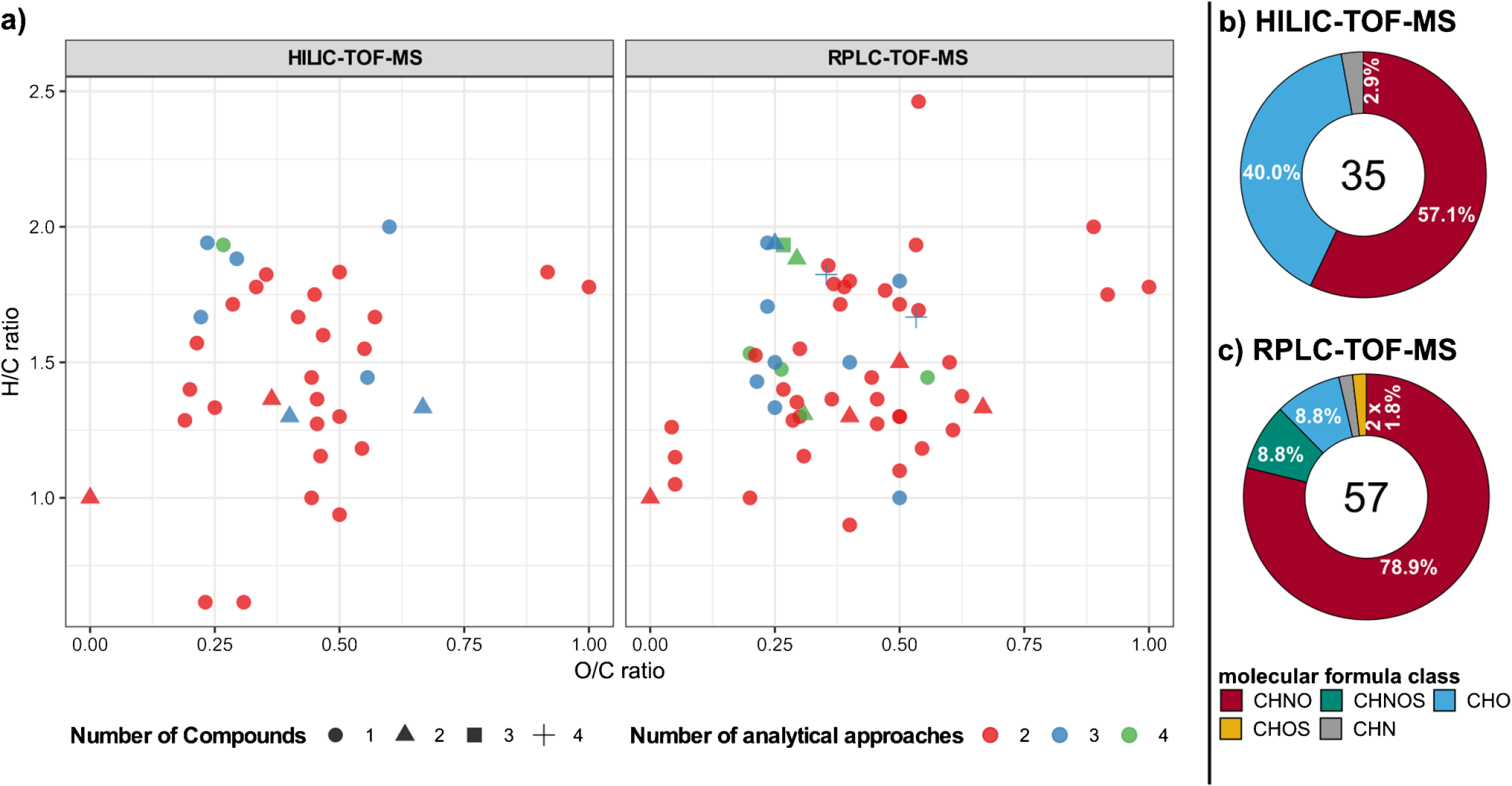
Van Krevelen diagram and molecular formula class for matches of FT-ICR-MS with RPLC-TOF-MS or HILIC-TOF-MS. **a** Color in the van Krevelen diagram indicates the number of analytical approaches by which a compound was detected (max. 4, min 2), different symbols indicate the number of compounds (retention times) separated by LC. **b** and **c** Relative ratios of molecular formula classes for matches of FT-ICR-MS with HILIC-TOF-MS or RPLC-TOF-MS (CHNO: red, CHNOS: cyan, CHO: blue, CHOS: yellow, CHN: gray). The number in the center of the charts is the total number of compounds

For 78 of the merged 92 *rth3*-mutant up-regulated molecular formulas, only one compound (showing only one RT within the entire chromatogram) was detected. Eleven molecular formulas showed two RT; one molecular formula three (C_15_H_29_N_3_O_4_), and two molecular formulas (C_15_H_25_N_3_O_8_, C_17_H_31_N_3_O_6_) appeared at four distinctive RT (Table S6, Fig. 5a). The three compounds detected at four and three RT, respectively, were compared with chemical formulas in the PubChem chemical database [73] resulting in possible tripeptides, e.g., Glu-Asp-Leu, Leu-Leu-Glu, or Leu-Leu-Ala, which are common metabolic intermediates and might be present in root exudates.

Of the 92 matched molecular formulas, 67 were detected with two analytical approaches, 18 molecular formulas with three, and 7 formulas with the maximum of 4 approaches (Table S6, Fig. 5a).

To compare the two analyzers (TOF-MS, FT-ICR-MS) from a more detailed, analytical perspective, we selected the raw intensities and *S/N* ratio of the top 15 compounds listed in Table S6 (SI Excel table, Tab: Data_Inten_compounds, Fig. S8). While the absolute intensity for the features is lower by several orders of magnitude for TOF-MS compared to FT-ICR-MS, the *S/N* ratio can be used for a comparison. Of the 22 features compared, 15 had a higher *S/N* ratio in the TOF-MS measurement. Normalization to the estimated amount of carbon injected for each feature lead to 12 features with a higher S/N ratio in the TOF-MS measurement.

Evidently, in DI-FT-ICR-MS, acquisition times of several minutes are applied to accumulate spectral signals via a constant sample intake, whereas in LC-TOF-MS, only a short transient signal can be monitored, which might have a negative impact on counting statistics in terms of S/N ratios. Besides the differences in sample preparation and thus in the matrix effects [74], the amount of carbon injected for the different approaches was comparable, i.e., RPLC-TOF-MS 1.7 µg, HILIC-TOF-MS 0.83 µg, and DI-FT-ICR-MS 1.25 µg carbon.

In conclusion, the sensitivities of the two platforms (TOF-MS, FT-ICR-MS) for the analysis of maize root exudates were similar (based on the common detected metabolites), and the differences in the exudation profile detected between the two analytical platforms were mostly related to the front-end separation and different sample preparation.

### Molecular characterization of compounds

The compounds detected jointly with RPLC-TOF-MS and FT-ICR-MS (*n* = 57) averaged at a similar O/C-ratio (0.40, *p*-value > 0.05) and H/C-ratio (1.50, *p*-value > 0.05) compared to those shared between HILIC-TOF-MS and FT-ICR-MS (O/C: 0.42, H/C: 1.47, *n* = 35). Also, compounds detected with RPLC-TOF-MS averaged at similar DBE (5.54, *p*-value > 0.05) and molecular mass (312 Da, *p*-value > 0.05) in comparison to HILIC-TOF-MS (DBE: 5.14, mass: 292 Da). *Kendrick* mass defect analysis showed more and longer CH_2_-homologue series with RPLC-TOF-MS (10 series with 2–4 homologues 33 molecular formulas without any CH_2_-homologue compared to the measurements with HILIC-TOF-MS (3 series with 2 homologues, 29 molecular formulas without any CH_2_-homologue) (data not shown).

Most compounds contained nitrogen and belonged to the CHNO formula class (Fig. 5b and c). The high abundance of nitrogen-containing compounds was independent of the LC-approach (Fig. S9), with RPLC-TOF-MS showing a higher fraction (51/57 molecular formulas, N/C-ratio 0.25 (*p*-value > 0.05)) compared to HILIC-TOF-MS (21/35 molecular formulas, N/C-ratio 0.20). Those compounds may be amino acids, nucleotides, or their derivatives (i.e., CHNO formula class), which are compounds detected with both chromatographic methods [75–77] and widely known to be present in root exudates samples [27, 77–79]. Together with amines and polyamines (i.e., CHN formula class), they represent a high percentage of exuded nitrogen-containing compounds [80]. For the RPLC-TOF-MS dataset, just 5 (8.8%) CHO-molecular formulas (i.e., do not contain any heteroatoms) were found, whereas HILIC-TOF-MS matches showed a higher abundance (14, 40%) of this molecular formulas class. These CHO-compounds are partly highly oxygenated (e.g., C_12_H_22_O_11_, C_9_H_16_O_9_), reflecting the capability of HILIC-TOF-MS to retain polar compounds such as sugars (i.e., CHO formula class). Just a few sulfur-containing compounds (6) were found, solely with RPLC-TOF-MS.

Comparison of the *rth3* up-regulated fraction of molecular formula classes detected solely with FT-ICR-MS (Fig. 4c) with the composition of the compounds (Fig. 5b and c) indicates a loss of CHO-formulas as well as a decreased fraction of the CHOS and CHNOS class, but an increase in the fraction of the CHNO class.

The high fraction of nitrogen containing compounds detected in this study was already observed in maize root exudates [38]. Nevertheless, some studies reported that the majority of exuded carbon, in terms of absolute quantity, belonged to the CHO class (65–73% sugars, 17–18% phenolics, 7–33% organic acids [81, 82]) and only a small fraction contributed to the CHNO class (2–3% amino acids [81, 82]). This suggests that other N-containing metabolites (e.g., growth factors, vitamins, and fatty acids, other secondary metabolites), while exuded in smaller quantities, might be responsible for the observed high molecular diversity in root exudates.

### Pathway matching

To highlight the link of the combined results with the maize plant metabolome, the compounds were assigned a KEGG ID by a query to the KEGG compound database based on the *rth3*-mutant up-regulated molecular formulas after RT-matching (from 92 molecular formulas, 75 were left after reprocessing sodium-adducts). While these resulted in 115 matches (i.e., KEGG IDs = metabolites), just 30 different molecular formulas could be found within these matches. For example, the molecular formula C_12_H_22_O_11_ matched 36 isomers in the KEGG database. Those matches include the diversity of the metabolome of multiple species and are not specific for *Zea mays* L. Therefore, additional filtering based on a species-specific pathway analysis was conducted.

Among the 115 KEGG IDs, 49 metabolites (43%) could be related to the metabolome of *Zea mays* L. (Table S7). Utilizing this annotation method based on molecular formulas, we can propose tentative structures for possible metabolites of *Zea mays* L. (identification confidence level 4 [23]). The assigned metabolites cover compound classes as present in maize root exudates such as sugars (e.g., sucrose, maltose, lactose), nucleosides (adenosine, guanine), and nucleobases (adenine, uracil) [83–85]. These 49 metabolites have just 21 different molecular formulas. As expected, the results indicate an overlap between root exudates and the metabolic pathways of *Zea mays* L. like the biosynthesis of secondary metabolites, the pyrimidine and purine metabolism, and the carbohydrate metabolism (Table S7).

A possible explanation for the high number of molecular formulas detected but not present in KEGG are (unknown) secondary metabolites not reported for the shoot metabolome. A specific database of compounds found in root exudates would benefit future studies on the characterization of root carbon input. Results of non-targeted (U)HRMS could be the basis of such a database since information of unknown compounds can be generated without available standards. It is apparent that most KEGG-matches have a low molecular mass (< 250 Da) and/or a high number of nitrogen (> 2) (Table S7). It appears that additional structural identification of root exudates with molecular masses > 250 Da is needed.

Although metabolite confirmation through chemical standards was not the aim of this study, the KEGG-identified compounds can be used for the validation of our presented approach and the molecular formulas found by confirming the link between exuded metabolites and the *Zea mays* L. metabolome. Nevertheless, MS/MS information and standards are needed further to confirm the chemical structure of the annotated molecular formulas and possibly identify compounds so far not reported to be present in the *Zea mays* L. metabolome.

## Conclusions

In this study, we used six different analytical approaches to reveal the molecular diversity of maize root exudates. The *rth3*-mutant genotypes showed a higher carbon and nitrogen exudation rate than their corresponding WT sibling despite the lack of root hairs. The higher *rth3* exudation trend was further confirmed from the metabolomics analyses, where, regardless of the analytical approach used, a higher number of features were revealed for the *rth3*-mutant. DI-FT-ICR-MS results showed a comparable number of detected molecular features as the LC-TOF-MS analysis, despite a different sample preparation approach and the absence of a front-end separation technique, and overall, 254 significant features were jointly detected with at least two analytical approaches based on molecular mass.

In non-targeted metabolomics studies, independently from the investigated sample type, compound coverage is often limited by ionization efficiency. For LC-TOF-MS measurements, the negative ionization resulted in more significant molecular features, whereas the opposite trend was observed for FT-ICR-MS results. However, for both mass spectrometric platforms, some molecular features were only detectable in one specific ionization, highlighting the importance of utilizing both ionization modes to increase metabolite coverage even for a single platform.

FT-ICR-MS can be a valuable tool to gain molecular insights into compound classes present in root exudates since molecular formulas can be assigned with high confidence in comparably short analysis time with similar sensitivity as compared to LC-TOF-MS. On the other hand, our results from root exudate samples underline that isomer separation via LC-TOF-MS is an essential prerequisite for metabolite identification and further pathway annotation. The combination of HILIC and RP with TOF-MS is able to fill the analytical window gap immanent to both methods and gives access to a comparable polarity space as DI-FT-ICR-MS.

The benefit of using both LC-TOF-MS and DI-FT-ICR-MS is the potential to annotate LC-TOF-MS derived masses and, based on molecular mass, add polarity information to direct infusion measurements. Despite the lack of RT data for DI-FT-ICR-MS and the lower mass accuracy and mass resolving power for LC-TOF-MS measurements typically hampering formulas annotation in the analysis of intact ions, the proposed workflow gives a direction on how to exploit and merge different mass spectrometric datasets to increase compound identification confidence and ultimately improve root exudate characterization. Overall, the small fraction of matches found between the FT-ICR-MS and TOF-MS platforms underline that a combination of two different MS-platforms can significantly improve the reliability of the results.

However, as there are often limitations in available instrumentation and measurement time, our results indicate that even with only one approach, root exudation trends can be successfully recovered. In such case, cautious treatment of background (blank) signals and other false positives by sample replication and strict quality control is crucial due to the missing validation of results with another approach in terms of intercomparison exercises.

In case isotope pattern matching is applied to validate non-targeted MS features, the S/N ratio of isotopologues may be too low, hence potentially eliminating otherwise significant features. Notably, for the matched significant features in LC-TOF-MS and DI-FT-ICR-MS, the S/N ratios were in a comparable range. However, in case of low abundance ions, DI-FT-ICR-MS does benefit from the potential of accumulating multiple scans to increase the S/N ratio, whereas any LC method may be limited by column loading.

Although we did not explicitly test DI-/ and LC-Orbitrap-MS, it can be expected that the discussed differences due to sample separation also apply to this mass analyzer and are more related to the chosen approach (i.e., DI or LC).

## Supplementary Information

Below is the link to the electronic supplementary material.

Supplementary file1 (DOCX 2.24 KB)

Supplementary file2 (XLSX 235 KB)

## Data Availability

The data for all significant and not significant molecular features/formulas for the exudate samples of all six analytical approaches used (RPLC-TOF-MS (+)/(−), HILIC-TOF-MS (+)/(−), and DI-ICR-MS (+)/(−)) is compiled here: SI Excel table, Tab: Data_all_features.
