# Supplementary material for: The effect of root hairs on exudate composition: a comparative non-targeted metabolomics approach
Source: Anal Bioanal Chem. 2022 Dec 22;415(5):823–40. doi: 10.1007/s00216-022-04475-9 (PMC9883335; doi:10.1007/s00216-022-04475-9)
Supplement: Supplementary file 1 — Supplementary file1 (DOCX 2.24 KB) [file 216_2022_4475_MOESM1_ESM.docx]

**Supporting Information**

**The effect of root hairs on exudate composition –**

**a comparative non-targeted metabolomics approach**

Martin Lohse^a#^; Michael Santangeli^b,c#^; Teresa Steininger-Mairinger^c^; Eva Oburger^b*^; Thorsten Reemtsma^a,d^; Oliver J. Lechtenfeld^a,e*^; Stephan Hann^c^

^a^ Department of Analytical Chemistry, Helmholtz Centre for Environmental Research – UFZ, 04318, Leipzig, Germany

^b^ Institute of Soil Research, Department of Forest and Soil Sciences, University of Natural Resources and Life Sciences, Vienna (BOKU), 3430, Tulln an der Donau, Austria.

^c^ Institute of Analytical Chemistry, Department of Chemistry, University of Natural Resources and Life Sciences, Vienna (BOKU), 1190, Vienna, Austria.

^d^ Institute of Analytical Chemistry, University of Leipzig, 04103, Leipzig, Germany

^e^ ProVIS – Centre for Chemical Microscopy, Helmholtz Centre for Environmental Research – UFZ, 04318, Leipzig, Germany

^#^ These authors contributed equally to this work.

* Corresponding Authors: [eva.oburger@boku.ac.at](mailto:eva.oburger@boku.ac.at); [oliver.lechtenfeld@ufz.de](mailto:oliver.lechtenfeld@ufz.de)

**Contents**

[Experimental details on the plant growth conditions, the analysis of chemical parameters, and the data processing for LC-TOF-MS and DI-FT-ICR-MS 3](#_Toc107070396)

[Plant growth conditions 3](#_Toc107070397)

[Analysis of chemical parameters 4](#_Toc107070398)

[Data processing LC-TOF-MS 5](#_Toc107070399)

[Data processing DI-FT-ICR-MS 6](#_Toc107070400)

[Additional Tables and Figures 8](#_Toc107070401)

[Table S1: Operational settings for the LC-TOF-MS system. 8](#_Toc107070402)

[Table S2: Summary chemical analysis maize root exudates. 9](#_Toc107070403)

[Table S3: Plant parameters for the plants sampled with the soil-hydroponics-hybrid approach. 10](#_Toc107070404)

[Table S4: Sample list of soil column (SC) used for each analytical approach for differential analysis. 11](#_Toc107070405)

[Figure S1: Workflow of differential statistical analysis used to screen significant molecular features in root exudates. 12](#_Toc107070406)

[Figure S2: Volcano plot FT-ICR-MS. 13](#_Toc107070407)

[Figure S3: Volcano plot HILIC-TOF-MS. 14](#_Toc107070408)

[Figure S4: Volcano plot RPLC-TOF-MS. 15](#_Toc107070409)

[Figure S5: Comparison of molecular formulas assigned for negative and positive ionization mode FT-ICR-MS measurements. 16](#_Toc107070410)

[Figure S6: Number of molecular formulas by the number of nitrogen atoms. 17](#_Toc107070411)

[Figure S7: RT vs mass plots of the different LC-TOF-MS approaches. 18](#_Toc107070412)

[Table S5: Number of pairwise, *rth3*-mutant up-regulated matches with FT-ICR-MS based on neutral mass. 19](#_Toc107070413)

[Table S6: Matched, significant, *rth3*-mutant up-regulated compounds based on neutral mass and retentions time. 20](#_Toc107070414)

[Figure S8: Heatmap for the comparison of the *S/N* ratio of matched, significant, *rth3*-mutant up-regulated compounds. 23](#_Toc107070415)

[Figure S9: Van Krevelen diagram for matches of FT-ICR-MS with RPLC-TOF-MS or HILIC-TOF-MS. 24](#_Toc107070416)

[Table S7: Result of KEGG-query: putative *Zea mays* metabolites. 25](#_Toc107070417)

[Supplemental References 31](#_Toc107070418)

Experimental details on the plant growth conditions, the analysis of chemical parameters, and the data processing for LC-TOF-MS and DI-FT-ICR-MS

Plant growth conditions

*Za mays* L. plants were grown for three weeks in a soil column experiment as described by Vetterlein et al. [1]. For the experiment, six biological replicates of each, the *Zea mays* L. root hair defective mutant (*rth3*) and the corresponding WT sibling were selected [2]. Transparent acrylic columns (250 mm height, 70 mm inner diameter, 5 mm wall thickness) were filled with loam soil to a height of 23 cm. A nylon mesh was placed at the bottom to retain the soil within the columns. The soil is a haplic Phaeozem from the vicinity of Schladebach in Saxony Anhalt, Germany (51°18’31.41” N; 12°6’16.31” E). The material was excavated from 0-50 cm depth and was used for the soil column experiment after sieving and homogenizing. The soil pH is 6.21 (in CaCl_2_), the total content of organic carbon is 8.4 mg g^-1^, and the texture is composed of 33.2% sand, 47.7% silt, and 19.1% clay [1].

The soil was sieved to < 1mm and fertilized by mixing with nutrients at the following concentrations (mg kg^−1^ soil): NH_4_NO_3_-N 50, K_2_SO_4_-K 50, MgCl_2_ 6 H_2_O-Mg 25, CaHPO_4_-P 40. The soil was then filled into the column by passing through a 2 mm sieve and compacted to a bulk density of 1.26 g cm^−3^ after filling. The columns were wrapped in aluminum foil to prevent algae growth. Maize seeds (*Zea mays* L. WT, and *rth3*-mutant) were surface sterilized in a 10% H_2_O_2_ solution, rinsed 4 times with MQW, and immersed in saturated CaSO_4_ solution for 3 h until planting. Seeds were planted at 1 cm depth within the substrate of the filled columns. The soil surface was covered by 40 g quartz gravel to minimize water evaporation. The columns were initially irrigated to a volumetric water content (VWC) of 22%. The columns were watered to initial water content every two days for 12 days and then daily until the end of the experiment.

The columns were placed in a climate chamber where growth conditions are controlled. The growing period was 21 days with exudate sampling and harvest on day 22. The photoperiod was set at 12h/12h. Light source intensity was 350 μmol m^−2^ s^-1^ of photosynthetically active radiation (PAR) at the top of the column. The temperature was kept at 22 °C during the day and 18 °C at night. Relative humidity was kept constant at 65%.

Analysis of chemical parameters

Dissolved organic carbon (DOC) and total nitrogen bound (TNb) concentrations were determined via high-temperature catalytic oxidation (Multi N/C 3100, Analytik Jena AG, Jena, Germany) after acidification of the samples to pH 2 with HCl. For the DOC the sampling blank was < LOQ (0.283 mg L^-1^), while for the TNb the sampling blank (0.14 mg L^-1^ *n* = 6) was subtracted from the concentrations. Carbon exudation rates were calculated based on root dry weight (dwt) and sampling time.

The pH-value of the undiluted exudates was determined with the pH-electrode Pro lab 4000 (SI analytics, Mainz, Germany) at 23 °C.

Anion analysis was performed via Ion Chromatography (Dionex™ ICS-6000 HPIC™

system, Thermo Fischer Scientific, Waltham, MA, USA). The separation was performed on an IonPac™ IC-column (Dionex™, AS18, 2 x 250 mm) using potassium

hydroxide as an eluent (generated via Dionex EGC 500 KOH Potassium Hydroxide

Eluent Generator Cartridge) at a flow rate of 0.25 mL min^−1^. Additionally, an AG18

Guard Column (2 x 50 mm) was used. A conductivity Detector (Dionex ICS-6000 CD)

was subsequently used for detection.

Data processing LC-TOF-MS

The Agilent MassHunter Profinder B.10.00 software (Agilent Technologies, Sta Clara, CA) was used for peak picking and chromatographic deconvolution with a Batch Recursive Feature Extraction (BRE, small molecules/peptides) workflow. The BRE consists of two stages: The *Molecular Feature Extraction* (MFE) and *Batch Find by Ion feature extraction* (FbI). MFE cleans data from background noise and summarizes all extracted features in each sample considering the mass accuracy, grouping of ions, isotopologue pattern, and the presence of different ion species or dimers [3], while FbI uses the median values derived from the MFE process to perform a targeted extraction to improve the reliability of data for differential analysis [4].

Thereafter the extracted features are aligned across all the samples according to *m/z* and retention time. To check the quality of the extracted features, a retention time and *m/z* window (±35 ppm; ±0.7 min) was set in the BRE to check the quality of the extracted feature on each sample, while the matching tolerance for mass accuracy and retention time were ±10 ppm and ±0.30 min, respectively.

Retention time extraction parameters were set at 2.00-14.00 min for RPLC-TOF-MS and 2.00-12.00 min for HILIC-TOF-MS. The *m/z* range was restricted to 100 -1 000 and only [M+H]^+^ and [M−H]^−^ ion species were allowed for positive and negative ionization respectively, while the maximum permitted charge state was two.

The quality of batch feature extraction is represented by the MFE and target (Tgt) scores, which are weighted averages reflecting the goodness of the software extracted features match with monoisotopic mass, isotopologue pattern, and retention time. The MFE and Tgt scores filters were set to ≥ 70, while the absolute peak height filter was set at 1 000 counts.

Following this, data were aligned in Agilent MassHunter Profiler Professional 15.0, alignment was performed according to RT and *m/z* similarities within the samples, without prior RT correction. Parameters applied for the alignment were the same as those defined previously during the extraction of features.

Data processing DI-FT-ICR-MS

FT-ICR-MS data was initially recorded in magnitude mode and later transformed to absorption mode using FTMSprocessing (v 2.2.0) [5]. An exudate pool sample was used to calibrate the phase equation that was subsequently applied to the other measurements. The full sine apodization function was applied by choosing the Kilgour function with the maximum occurring at half of the original transient length (F = 0.5) resulting in a mass resolving power of 1 200 000 at *m/z* 341.

Internal re-calibration of averaged spectra was done with a list of masses of sugars and background organic matter peaks and their respective sodium or chlorine adducts (pos. mode *m/z* 165–689, *n* = 22; neg. mode *m/z* 87–827, *n* = 62, linear calibration function). The root mean square error (RMSE) of the calibration masses was below 0.2 ppm. Peaks were considered detected if the signal-to-noise (*S/N*) ratio was greater than two. Raw spectra were processed with Compass DataAnalysis 5.0 (Bruker Daltonics, MA, U.S.A.).

Molecular formulas were assigned to peaks in the range 74-1 000 *m/z* allowing for elemental compositions C_1-80_ H_0-198_ O_0-40_ N_0-5_ S_0-3_ for positive mode additional Na_0–1_ with an error range of ± 0.5 ppm according to Lechtenfeld et al. [6] and Koch et al. [7]. Briefly, the following rules were applied: 0.3 ≤ H/C ≤ 2.5, 0 ≤ O/C ≤ 1.2, 0 ≤ N/C ≤ 1.5, 0 ≤ double bond equivalent (DBE) ≤ 25 (double bound equivalent, DBE = 1 + 1/2 (2C − H + N), Koch et al.), [8] −10 ≤ DBE-O ≤ 10 (Herzsprung et al.[9]), and element probability rules proposed by Kind and Fiehn [10]. Molecular formulas containing N_5_S_2_ and N_5_S_3_ were removed from the dataset due to possible false assignment of ^13^C isotopologues of CHO formulas [11].

Isotope formulas were removed from the dataset as they represent duplicate chemical information. The mass error range in the final dataset was limited to the 5^th^–95^th^ percentile of errors of CHO formulas in the initial dataset (neg. mode ± 0.384 ppm, pos. mode ± 0.466 ppm). For the positive ionization mode measurement a high number of multiple assignments, mainly for the CHOS and CHONS molecular formula class, were generated. For this reason, only molecular formulas were further processed when not multiple molecular formulas were assigned to one accurate mass.

Since the absorption mode processing allowed for more sensitive detection of mass peaks a lower *S/N* ratio for peak picking could be selected. To circumvent peak detection being influenced based on different dilution factors, a S/N ratio of 5 was used as a lower limit for all DI-FT-ICR-MS measurements and the dilution factor was later included for the normalization (see chapter on statistical analysis).

Aggregated molecular descriptors (formula mass, O/C-ratio, H/C-ratio, N/C-ratio, DBE) were calculated as number-based mean values, without using molecular mass as weighting factor. The H/C-ratio and DBE refers to the degree of unsaturation. The O/C-ratio indicates oxygenation and thus polarity of a compound, and similarly to the N/C-ratio illustrates the involvement of heteroatoms.

Additional Tables and Figures

Table S1: Operational settings for the LC-TOF-MS system.

| **LC** | **RPLC** | **HILIC** |
| --- | --- | --- |
| Stationary phase | 150 x 2.1 mm Atlantis^®^ T3 C-18, 3 µm particle size | 150 x 2.1 mm X-Bridge amide, 3.5 µm particle size |
| Mobile phase A | 99.9% v/v H_2_O, 0.1% v/v HCOOH | 10mM Ammonium Formate, pH 3.0 |
| Mobile phase B | 100% Methanol | 99.9% v/v ACN, 0.1% v/v HCOOH |
| Injection volume (µL) | 5 | 5 |
| Flow rate (µL min^-1^) | 200 | 200 |
| Column temperature (°C) | 40 | 40 |
| **ESI-TOF-MS** |  |  |
| Gas Temperature (°C) | 225 | 280 |
| Drying gas Flow (L min^-1^) | 8 | 5 |
| Nebulizer pressure (psi) | 30 | 35 |
| Capillary voltage (V) | 3500 | 3500 |
| Skimmer voltage (V) | 65 | 65 |
| Fragmentor voltage (V) | 120 | 120 |

Table S2: Summary chemical analysis maize root exudates.

Dilution factor for DI-FT-ICR-MS measurement to get to a 5 mg L^-1^ DOC, TNb after blank subtraction: (0.14 mg L^-1^ n = 6). Nitrate was below the limit of detection in all samples and blanks ( < 0.05 mg L^-1^). Soil column 3 was excluded from any statistical analysis.

| **column number** | **maize genotype** | **DOC [mg L^-1^]** | **TN_b_ [mg L^-1^]** | **Chloride [mg L^-1^]** | **Sulfate [mg L^-1^]** | **pH** | **Dilution factor** |
| --- | --- | --- | --- | --- | --- | --- | --- |
| 1 | WT | 23.67 | 0.74 | 0.15 | 0.57 | 6.17 | 4.73 |
| 2 | rth3 | 14.60 | 0.84 | 0.26 | 0.43 | 6.17 | 2.92 |
| 3 | WT | 11.89 | 0.60 | 0.13 | 0.37 | 6.07 | 2.38 |
| 4 | rth3 | 14.82 | 0.79 | 0.18 | 0.40 | 6.07 | 2.96 |
| 5 | WT | 22.49 | 0.74 | 0.22 | 0.52 | 6.30 | 4.50 |
| 6 | rth3 | 22.31 | 1.21 | 0.13 | 0.40 | 6.06 | 4.46 |
| 7 | WT | 22.27 | 0.82 | 0.18 | 0.51 | 6.44 | 4.45 |
| 8 | rth3 | 12.76 | 0.89 | 0.17 | 0.33 | 5.94 | 2.55 |
| 9 | WT | 18.89 | 0.88 | 0.15 | 0.47 | 6.07 | 3.78 |
| 10 | rth3 | 23.93 | 0.79 | 0.14 | 0.36 | 6.32 | 4.79 |
| 11 | WT | 28.44 | 0.87 | 0.17 | 0.59 | 6.29 | 5.69 |
| 12 | rth3 | 15.73 | 0.87 | 0.16 | 0.36 | 6.13 | 3.15 |

Table S3: Plant parameters for the plants sampled with the soil-hydroponics-hybrid approach.

Soil column 3 was excluded from any statistical analysis.

| **column number** | **maize genotype** | **fresh weight leaf 4 [g]** | **dry weight leaf 4 [g]** | **fresh weight shoot [g]** | **dry weight shoot [g]** | **fresh weight roots [g]** | **dry weight roots [g]** |
| --- | --- | --- | --- | --- | --- | --- | --- |
| 1 | WT | 1.45 | 0.1 | 18.32 | 2.09 | 17.77 | 1.71 |
| 2 | rth3 | 1.04 | 0.02 | 9.65 | 0.82 | 10.23 | 0.63 |
| 3 | WT | 1.15 | 0.01 | 10.91 | 0.95 | 8.21 | 0.62 |
| 4 | rth3 | 1.15 | 0.01 | 9.86 | 0.86 | 9.13 | 0.62 |
| 5 | WT | 1.32 | 0.05 | 19.10 | 2.25 | 16.92 | 1.64 |
| 6 | rth3 | 1.12 | 0.01 | 12.58 | 2.18 | 13.01 | 1.08 |
| 7 | WT | 1.38 | 0.07 | 15.49 | 1.70 | 15.81 | 2.12 |
| 8 | rth3 | 0.96 | 0.02 | 7.95 | 0.65 | 8.85 | 0.76 |
| 9 | WT | 1.40 | 0.07 | 16.13 | 1.73 | 16.87 | 2.49 |
| 10 | rth3 | 1.09 | 0.05 | 11.90 | 1.09 | 11.94 | 0.94 |
| 11 | WT | 1.45 | 0.08 | 16.79 | 2.07 | 18.98 | 2.29 |
| 12 | rth3 | 1.08 | 0.04 | 10.10 | 0.93 | 10.92 | 0.97 |

Table S4: Sample list of soil column (SC) used for each analytical approach for differential analysis.

| **Analytical approach** | **Samples** |
| --- | --- |
| DI-FT-ICR-MS | WT (SC 1, 5, 7, 9, 11)^†^  *rth3* (SC 2, 4, 6, 8, 10, 12)  Blank (1, 2, 3, 4, 5, 6) |
| HILIC-TOF-MS | WT (SC 1, 5, 7, 11)^†^*;  *rth3* (SC 2, 4, 6, 8, 10, 12)  Blank (1, 2, 3, 4, 5, 6) |
| RPLC-TOF-MS | WT (SC 1, 5, 7, 11) ^†^*;  *rth3* (SC 2, 4, 6, 8, 10, 12)^##^  Blank (1, 3, 4, 5, 6)^#^ |

†SC3 was excluded from the workflow due to significantly different biomass compared to the other replicates.

*SC 9 was lost during LC-TOFMS sample preparation.

^#^ Blank 2 was lost during RPLC-TOF-MS sample preparation.

^##^ SC 2 was removed from RPLC-TOF-MS (-) due to contamination before injection.


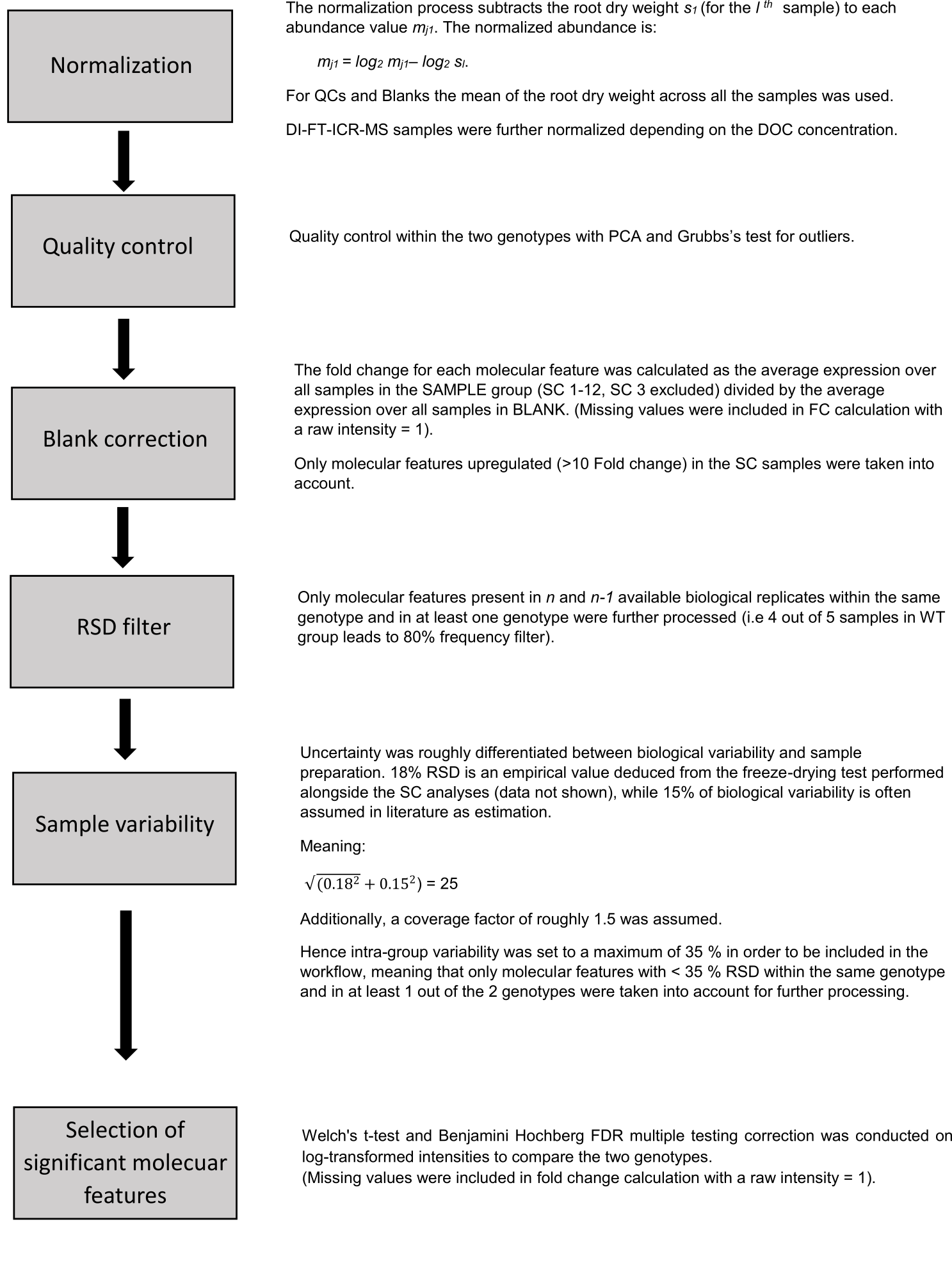


Figure S1: Workflow of differential statistical analysis used to screen significant molecular features in root exudates.


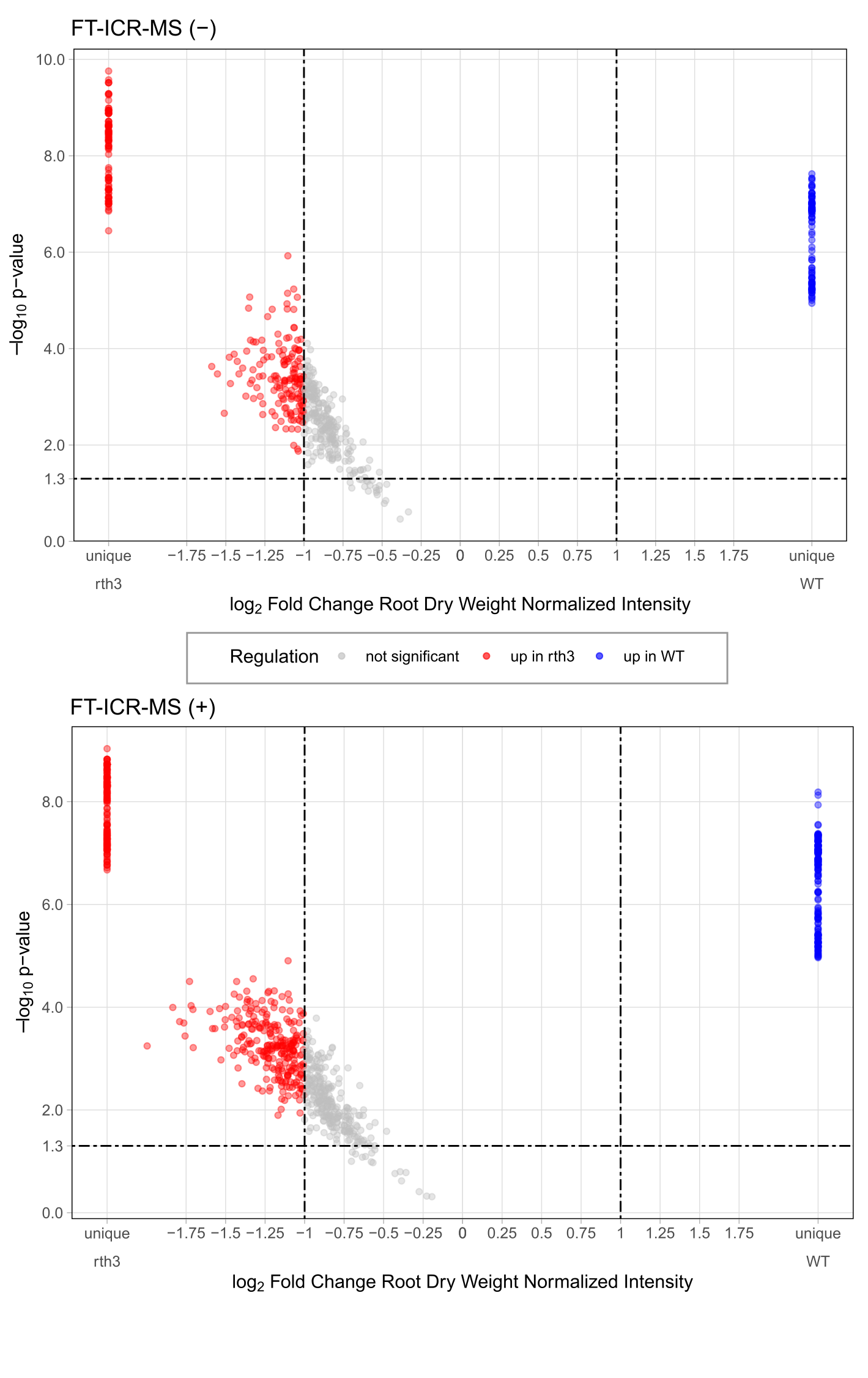


Figure S2: Volcano plot FT-ICR-MS.

Molecular formulas up-regulated in WT and the *rth3*-mutant are shown as blue and red dots, respectively. Not significant molecular formulas are represented as grey dots. The p-value threshold (0.05) and FC threshold (>2) lines are shown in the graphs as dashed lines.


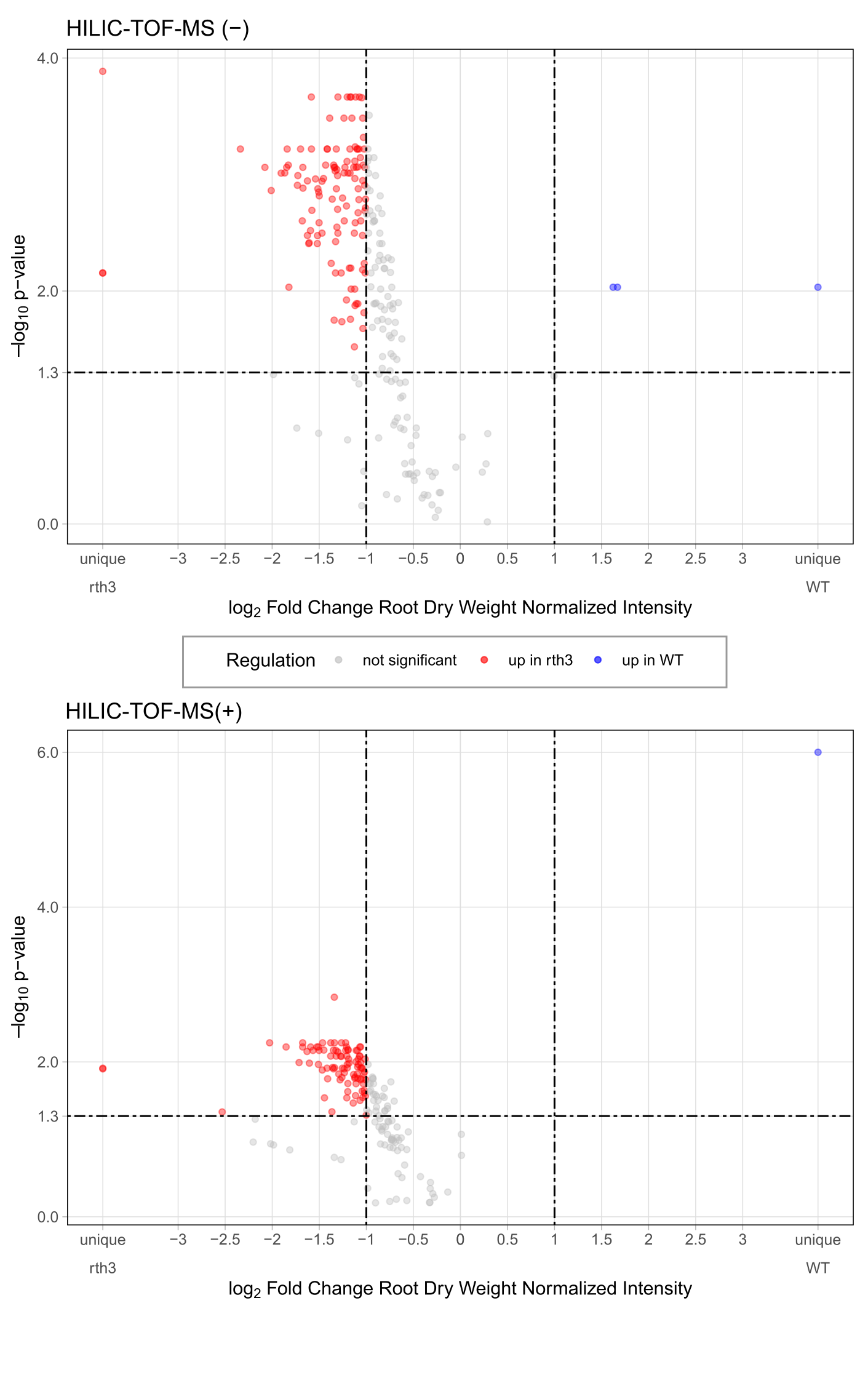


Figure S3: Volcano plot HILIC-TOF-MS.

Molecular features up-regulated in WT and the *rth3*-mutant are shown as blue and red dots, respectively. Not significant molecular features are represented as grey dots. The p-value threshold (0.05) and FC threshold (>2) lines are shown in the graphs as dashed lines.


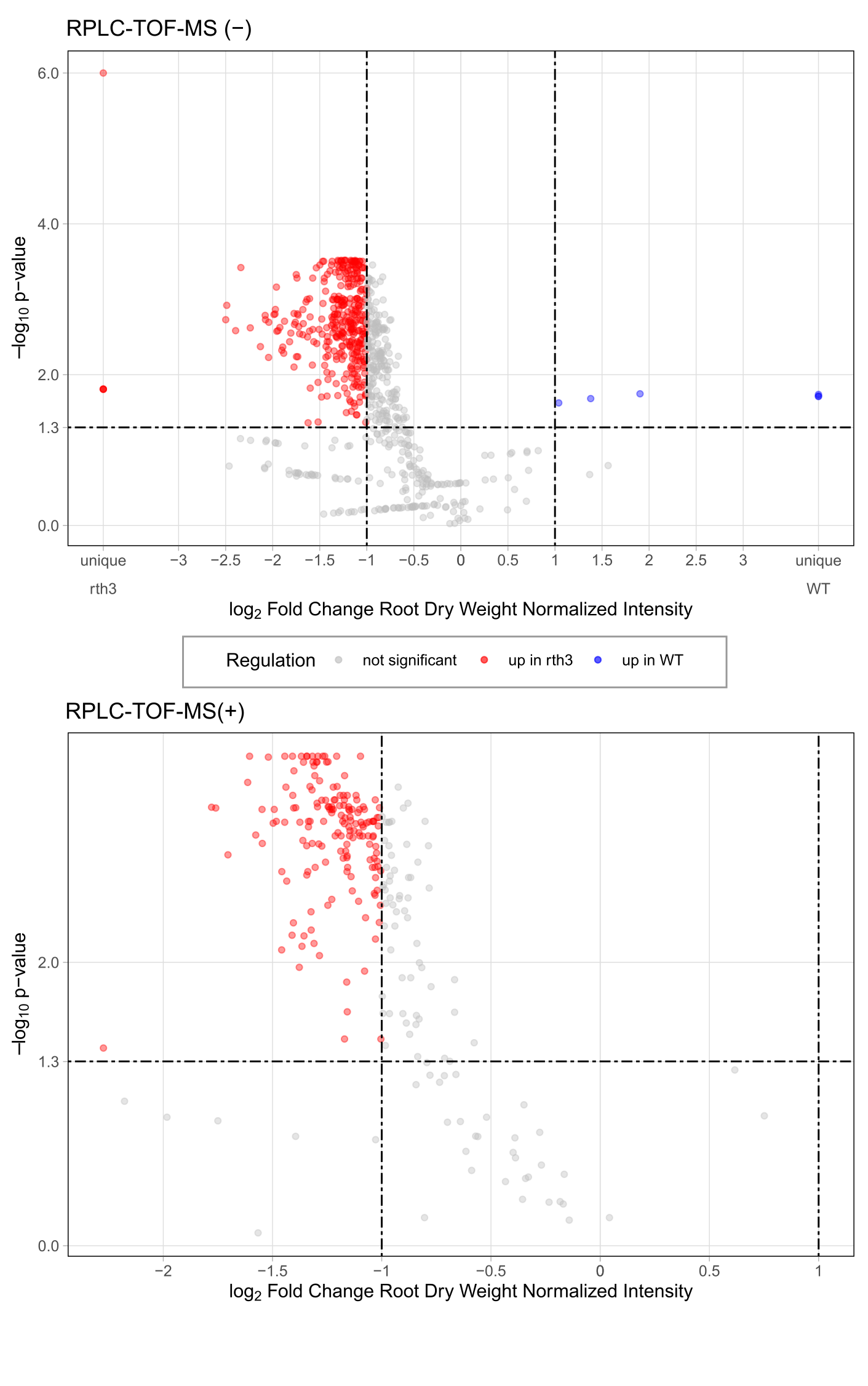


Figure S4: Volcano plot RPLC-TOF-MS.

Molecular features up-regulated in WT and the *rth3*-mutant are shown as blue and red dots, respectively. Not significant molecular features are represented as grey dots. The p-value threshold (0.05) and FC threshold (>2) lines are shown in the graphs as dashed lines.


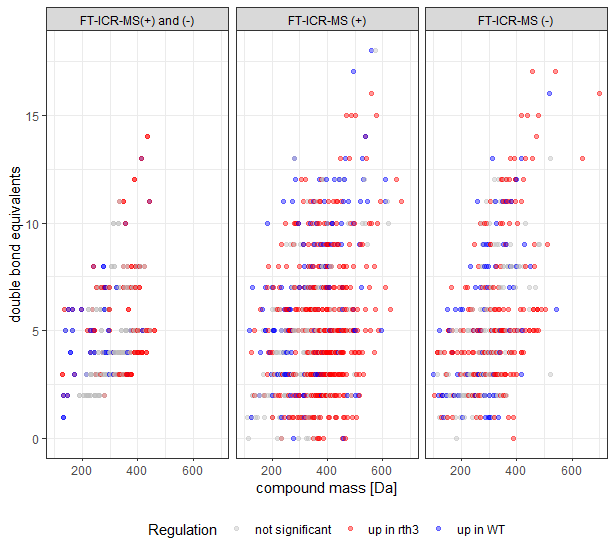


Figure S5: Comparison of molecular formulas assigned for negative and positive ionization mode FT-ICR-MS measurements.

Double bound equivalent vs mass diagram for comparison of molecular formulas assigned for negative mode and positive mode FT-ICR-MS measurements.


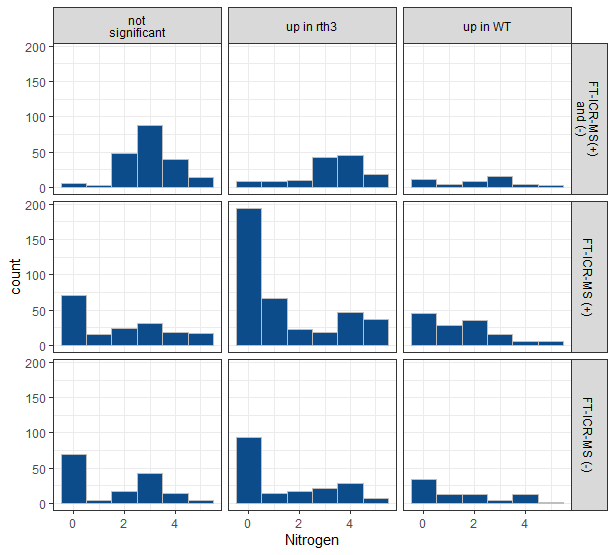


Figure S6: Number of molecular formulas by the number of nitrogen atoms.

The absolute number of molecular formula by the number of nitrogen atoms (none - up to 5) for the up-regulated and non-significant compounds as detected with either DI-FT-ICR-MS (+), DI-FT-ICR-MS (−), or jointly with the two ionization modes.


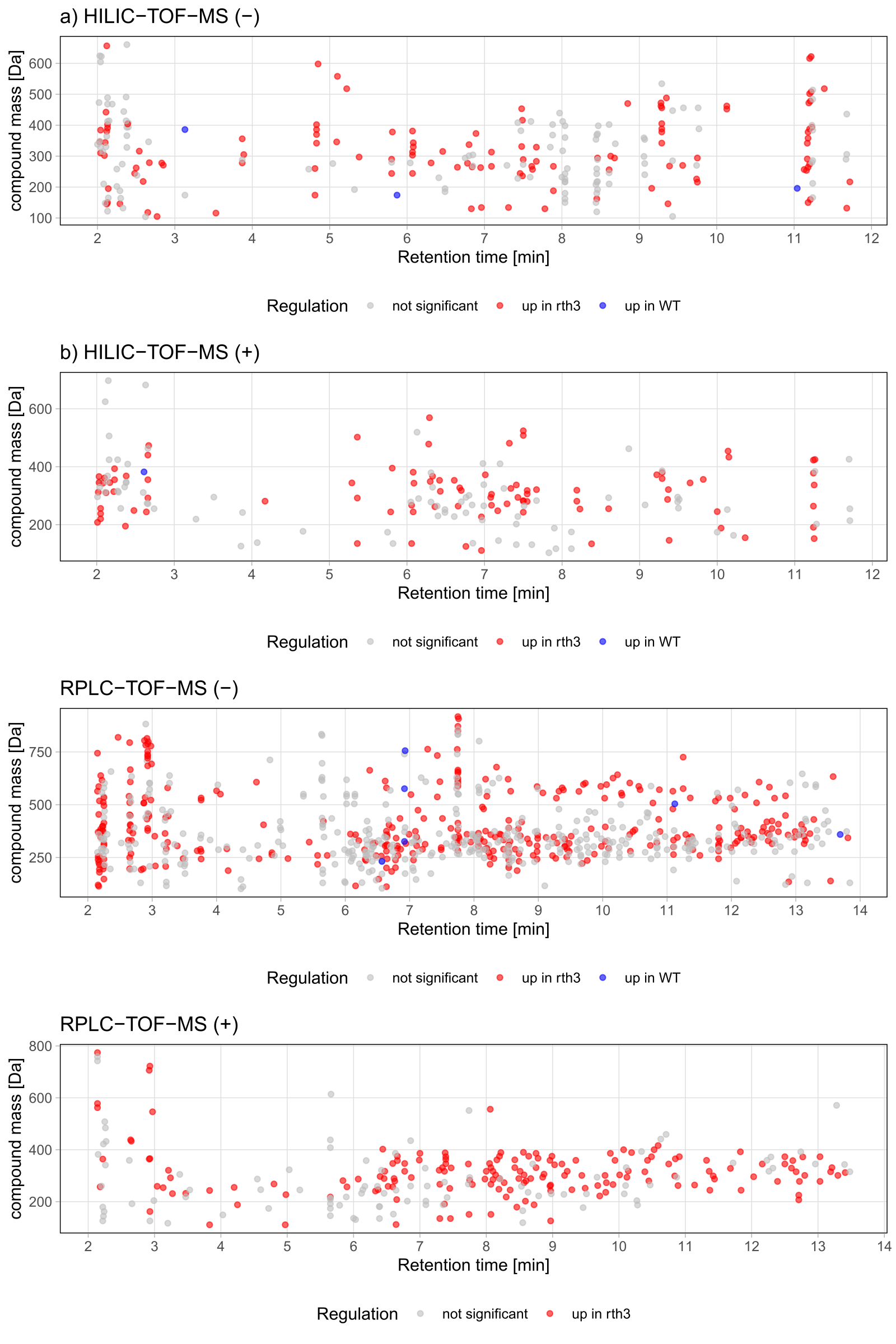


Figure S7: RT vs mass plots of the different LC-TOF-MS approaches.

- Molecular features up-regulated in WT and the *rth3*-mutant are shown as blue and red dots, respectively. Not significant molecular features are represented as grey dots

Table S5: Number of pairwise, *rth3*-mutant up-regulated matches with FT-ICR-MS based on neutral mass.

| **pairwise match between:** | | **Number of matches** |
| --- | --- | --- |
| FT-ICR-MS (+)* | FT-ICR-MS (−)* | 38 |
| RPLC-TOF-MS (−) | FT-ICR-MS (−) | 36 |
| RPLC-TOF-MS (+) | FT-ICR-MS (−) | 29 |
| RPLC-TOF-MS (+) | FT-ICR-MS (+) | 24 |
| RPLC-TOF-MS (−) | FT-ICR-MS (+) | 19 |
| HILIC-TOF-MS (+) | FT-ICR-MS (+) | 16 |
| HILIC-TOF-MS (−) | FT-ICR-MS (−) | 15 |
| HILIC-TOF-MS (+) | FT-ICR-MS (−) | 11 |
| HILIC-TOF-MS (−) | FT-ICR-MS (+) | 7 |

* Only 9 matched molecular formulas were found to be unique for the WT (i.e. these were not observed in the *rth3*-mutant) as a result of the FT-ICR-MS (+) and FT-ICR-MS (−) matching: C_6_H_13_NO_2_, C_6_H_9_N_3_O_2_, C_10_H_17_N_3_O_4_, C_12_H_21_N_3_O_5_, C_14_H_17_N_3_O_3_, C_14_H_20_N_2_O_4_, C_16_H_28_N_4_O_6_, C_18_H_30_O_2_, C_18_H_32_O_3_. Those molecular formulas were neglected during the subsequent data analysis since they had no overlap with any LC-TOF-MS approach.

Table S6: Matched, significant, *rth3*-mutant up-regulated compounds based on neutral mass and retentions time.

Table sorted by an increasing number of carbon atoms: neutral molecular formula and exact mass, the LC approach in which the mass of the molecular formula was detected, and the respective retentions times for the positive (+) and negative (−) ionization polarity. The column *overlap (+) and (−)* contains the retention time when a compound was detected. If within a 0.1 min RT window between the positive (+) and negative (−) measurements the same mass was detected, the mean of the two RT was calculated. The number of RT in this column equals the number of compounds. If a molecular formula was detected with DI-FT-ICR-MS (FT (+) or FT (−)) the value 1 is shown in the table. Number approaches indicate how many analytical approaches detected a certain mass (maximum 4) the number of different KEGG-IDs (metabolites, in total 49) is given for a specific molecular formula after matching with metabolites present in *Zea mays (*zma*)* metabolic pathways [12]. The KEGG-IDs and further information on the matches are shown in Table S7.

| **#** | **Molecular formula** | **Exact, neutral mass of molecular formula** | **LC approach** | **RT (min): (−)** | **RT (min): (+)** | **Overlap (+) and (−)** | **FT (+)** | **FT (−)** | **Number ap-proches** | **Number KEGG-ID’s**  **(zma)** |
| --- | --- | --- | --- | --- | --- | --- | --- | --- | --- | --- |
| 1 | C4H4N2O2 | 112.02728 | RPLC | 6.64 | 6.64 | 6.64 |  | 1 | 3 | 1 |
| 2 | C5H10N2O3 | 146.06914 | HILIC | 9.37 | 9.38 | 9.38 |  | 1 | 3 | 4 |
| 3 | C5H5N5 | 135.05450 | HILIC |  | 5.36, 6.06 | 5.36, 6.06 | 1 |  | 2 | 1 |
| 4 | C5H5N5 | 135.05450 | RPLC |  | 7.3, 7.46 | 7.3, 7.46 | 1 |  | 2 | 1 |
| 5 | C5H5N5O | 151.04941 | RPLC |  | 8.07 | 8.07 |  | 1 | 2 | 1 |
| 6 | C5H7N3O | 125.05891 | HILIC |  | 6.76 | 6.76 | 1 |  | 2 | 1 |
| 7 | C7H9NO2 | 139.06333 | RPLC | 13.54 |  | 13.54 |  | 1 | 2 |  |
| 8 | C8H11NO5 | 201.06372 | RPLC | 6.64 |  | 6.64 |  | 1 | 2 |  |
| 9 | C9H12N2O6 | 244.06954 | HILIC | 5.8, 7.46 | 5.79 | 5.8, 7.46 |  | 1 | 3 | 2 |
| 10 | C9H12N2O6 | 244.06954 | RPLC | 4.66, 6.65 |  | 4.66, 6.65 |  | 1 | 2 | 2 |
| 11 | C9H13N3O4 | 227.09061 | HILIC |  | 6.96 | 6.96 |  | 1 | 2 | 1 |
| 12 | C9H13N3O4 | 227.09061 | RPLC |  | 4.98 | 4.98 |  | 1 | 2 | 1 |
| 13 | C9H13N3O5 | 243.08552 | HILIC |  | 7.5 | 7.5 | 1 | 1 | 3 | 2 |
| 14 | C9H13N3O5 | 243.08552 | RPLC | 3.76 | 3.83 | 3.8 | 1 | 1 | 4 | 2 |
| 15 | C9H16O9 | 268.07943 | HILIC | 9.39 |  | 9.39 |  | 1 | 2 | 2 |
| 16 | C9H16O9 | 268.07943 | RPLC | 2.67 |  | 2.67 |  | 1 | 2 | 2 |
| 17 | C9H18O8 | 254.10017 | RPLC | 8.25 |  | 8.25 |  | 1 | 2 | 1 |
| 18 | C9H9NO4 | 195.05316 | HILIC |  | 2.37 | 2.37 |  | 1 | 2 | 3 |
| 19 | C10H11NO5 | 225.06372 | RPLC |  | 12.71 | 12.71 |  | 1 | 2 | 2 |
| 20 | C10H13N2NaO5 | 264.07222 | RPLC |  | 8.97 | 8.97 | 1 |  | 2 | 2 |
| 21 | C10H13N5O3 | 251.10184 | RPLC |  | 7.45 | 7.45 | 1 |  | 2 | 1 |
| 22 | C10H13N5O4 | 267.09675 | HILIC | 7.09 | 6.06, 7.09 | 6.06, 7.09 | 1 |  | 3 | 2 |
| 23 | C10H13N5O4 | 267.09675 | RPLC |  | 7.3, 8.07 | 7.3, 8.07 | 1 |  | 2 | 2 |
| 24 | C10H13N5O5 | 283.09167 | HILIC | 7.67 |  | 7.67 |  | 1 | 2 | 1 |
| 25 | C10H13N5O5 | 283.09167 | RPLC | 7.75 |  | 7.75 |  | 1 | 2 | 1 |
| 26 | C10H15N3O4 | 241.10626 | RPLC | 6.31 | 6.33 | 6.32 |  | 1 | 3 | 0 |
| 27 | C10H15N3O5 | 257.10117 | RPLC |  | 2.18, 5.9 | 2.18, 5.9 |  | 1 | 2 | 0 |
| 28 | C10H15N3O6 | 273.09609 | RPLC | 4.86 |  | 4.86 |  | 1 | 2 | 0 |
| 29 | C10H18N2O5 | 246.12157 | RPLC | 9.27 | 9.29 | 9.28 | 1 |  | 3 | 3 |
| 30 | C10H9NO4 | 207.05316 | RPLC |  | 12.71 | 12.71 | 1 |  | 2 | 2 |
| 31 | C11H13NO6 | 255.07429 | HILIC |  | 8.6 | 8.6 | 1 |  | 2 | 0 |
| 32 | C11H13NO6 | 255.07429 | RPLC |  | 4.2 | 4.2 | 1 |  | 2 | 0 |
| 33 | C11H14N2O5 | 254.09027 | HILIC |  | 8.23 | 8.23 | 1 |  | 2 | 0 |
| 34 | C11H14N2O5 | 254.09027 | RPLC |  | 3.13 | 3.13 | 1 |  | 2 | 0 |
| 35 | C11H15N5O4 | 281.11240 | HILIC |  | 4.17, 8.19 | 4.17, 8.19 | 1 |  | 2 | 0 |
| 36 | C11H15N5O4 | 281.11240 | RPLC |  | 5.84 | 5.84 | 1 |  | 2 | 0 |
| 37 | C11H15N5O5 | 297.10732 | HILIC | 5.38 |  | 5.38 | 1 |  | 2 | 0 |
| 38 | C11H15N5O5 | 297.10732 | RPLC | 7.48 |  | 7.48 | 1 |  | 2 | 0 |
| 39 | C12H16N2O3 | 236.11609 | HILIC | 7.49 |  | 7.49 |  | 1 | 2 | 0 |
| 40 | C12H16N2O3 | 236.11609 | RPLC | 9.76 | 9.8 | 9.78 |  | 1 | 3 | 0 |
| 41 | C12H20O5 | 244.13107 | HILIC | 2.48 |  | 2.48 |  | 1 | 2 | 0 |
| 42 | C12H21NaO11 | 364.09816 | RPLC |  | 2.92 | 2.92 | 1 |  | 2 | 0 |
| 43 | C12H22O11 | 342.11621 | HILIC | 9.29 |  | 9.29 |  | 1 | 2 | 10 |
| 44 | C12H22O6 | 262.14164 | HILIC | 2.5 |  | 2.5 |  | 1 | 2 | 0 |
| 45 | C13H15N3O4S2 | 341.05040 | RPLC | 7.77 |  | 7.77 |  | 1 | 2 | 0 |
| 46 | C13H15NaO6 | 290.07663 | HILIC | 5.8 |  | 5.8 | 1 |  | 2 | 0 |
| 47 | C13H17N3O4 | 279.12191 | RPLC | 9.48 | 6.62 | 6.62, 9.48 | 1 | 1 | 4 | 0 |
| 48 | C13H22N4O7 | 346.14885 | RPLC | 12.98 |  | 12.98 |  | 1 | 2 | 0 |
| 49 | C13H32N4O7S | 388.19917 | RPLC |  | 7.38 | 7.38 |  | 1 | 2 | 0 |
| 50 | C13H8N5NaO3 | 305.05248 | HILIC |  | 7.09 | 7.09 | 1 |  | 2 | 0 |
| 51 | C13H8N5NaO4 | 321.04740 | HILIC |  | 7.67 | 7.67 | 1 |  | 2 | 0 |
| 52 | C14H20N2O3S | 296.11946 | RPLC | 11.99 | 12.03 | 12.01 | 1 |  | 3 | 0 |
| 53 | C14H22O3 | 238.15689 | HILIC |  | 2.05 | 2.05 |  | 1 | 2 | 0 |
| 54 | C14H24N4O7 | 360.16450 | RPLC | 6.98 |  | 6.98 |  | 1 | 2 | 0 |
| 55 | C14H24O4 | 256.16746 | HILIC |  | 2.04 | 2.04 |  | 1 | 2 | 0 |
| 56 | C14H26N4O5 | 330.19032 | RPLC |  | 7.47 | 7.47 |  | 1 | 2 | 0 |
| 57 | C15H21N3O4 | 307.15321 | RPLC | 10.74 |  | 10.74 |  | 1 | 2 | 0 |
| 58 | C15H23N3O3 | 293.17394 | RPLC | 6.81 | 6.87 | 6.84 | 1 | 1 | 4 | 0 |
| 59 | C15H24O7 | 316.15220 | HILIC | 2.54 |  | 2.54 |  | 1 | 2 | 0 |
| 60 | C15H25N3O8 | 375.16416 | RPLC | 8.18 | 6.66, 8.2, 8.64, 8.99 | 6.66, 8.19, 8.64, 8.99 |  | 1 | 3 | 0 |
| 61 | C15H27N5O6 | 373.19613 | RPLC |  | 7.39 | 7.39 |  | 1 | 2 | 0 |
| 62 | C15H29NO8 | 351.18932 | RPLC | 13.13 |  | 13.13 | 1 |  | 2 | 0 |
| 63 | C15H29N3O4 | 315.21581 | HILIC | 6.46 | 6.43 | 6.44 | 1 | 1 | 4 | 0 |
| 64 | C15H29N3O4 | 315.21581 | RPLC | 12.63, 13.13 | 10.39, 12.67, 13.19 | 10.39, 12.65, 13.16 | 1 | 1 | 4 | 0 |
| 65 | C16H15NO8 | 349.07977 | HILIC |  | 6.3 | 6.3 |  | 1 | 2 | 0 |
| 66 | C16H31N3O4 | 329.23146 | RPLC | 11.82, 12.57 | 12.6 | 11.82, 12.59 | 1 |  | 3 | 0 |
| 67 | C17H23N3O5 | 349.16377 | RPLC | 11.38 |  | 11.38 |  | 1 | 2 | 0 |
| 68 | C17H29N5O4 | 367.22195 | RPLC |  | 8.78 | 8.78 | 1 | 1 | 3 | 0 |
| 69 | C17H30N4O8 | 418.20636 | RPLC | 8.73 |  | 8.73 |  | 1 | 2 | 0 |
| 70 | C17H31N3O6 | 373.22129 | HILIC | 6.89 |  | 6.89 |  | 1 | 2 | 0 |
| 71 | C17H31N3O6 | 373.22129 | RPLC | 10.47, 10.89, 12.48, 12.99 | 10.49, 10.91, 12.5, 13.03 | 10.48, 10.9, 12.49, 13.01 |  | 1 | 3 | 0 |
| 72 | C17H32N4O5 | 372.23727 | HILIC |  | 7.01 | 7.01 | 1 | 1 | 3 | 0 |
| 73 | C17H32N4O5 | 372.23727 | RPLC | 12.25, 12.73 | 12.77 | 12.25, 12.75 | 1 | 1 | 4 | 0 |
| 74 | C17H33N3O4 | 343.24711 | HILIC | 6.08 |  | 6.08 | 1 | 1 | 3 | 0 |
| 75 | C17H33N3O4 | 343.24711 | RPLC | 13.81 |  | 13.81 | 1 | 1 | 3 | 0 |
| 76 | C18H30O4 | 310.21441 | HILIC | 2.04 | 2.12 | 2.08 |  | 1 | 3 | 4 |
| 77 | C18H32N4O7 | 416.22710 | RPLC |  | 10.59 | 10.59 |  | 1 | 2 | 0 |
| 78 | C18H32O6 | 344.21989 | HILIC | 2.1 |  | 2.1 |  | 1 | 2 | 0 |
| 79 | C19H28N4O5 | 392.20597 | RPLC | 11.81 | 11.83 | 11.82 | 1 | 1 | 4 | 0 |
| 80 | C19H29N3O4 | 363.21581 | RPLC | 13.22 |  | 13.22 |  | 1 | 2 | 0 |
| 81 | C19H34N4O7 | 430.24275 | RPLC | 13.28 |  | 13.28 |  | 1 | 2 | 0 |
| 82 | C20H21NaOS2 | 364.09315 | RPLC |  | 2.22 | 2.22 | 1 |  | 2 | 0 |
| 83 | C20H23N3OS | 353.15618 | RPLC | 8.72 |  | 8.72 |  | 1 | 2 | 0 |
| 84 | C20H30N4O5 | 406.22162 | RPLC | 13.09 |  | 13.09 | 1 | 1 | 3 | 0 |
| 85 | C20H31NaO11 | 470.17641 | HILIC | 8.85 |  | 8.85 | 1 |  | 2 | 0 |
| 86 | C20H31NaO6 | 390.20183 | RPLC | 8.54 |  | 8.54 | 1 |  | 2 | 3 |
| 87 | C20H35NaO9 | 442.21788 | HILIC | 2.11 |  | 2.11 | 1 |  | 2 | 0 |
| 88 | C21H27NaO4 | 366.18070 | HILIC |  | 2.03 | 2.03 | 1 |  | 2 | 0 |
| 89 | C21H35NaO12 | 502.20262 | HILIC |  | 5.36 | 5.36 | 1 |  | 2 | 0 |
| 90 | C21H36N5NaO8 | 509.24616 | RPLC | 12.39 |  | 12.39 | 1 |  | 2 | 0 |
| 91 | C23H29N3OS | 395.20313 | RPLC | 10.04 |  | 10.04 |  | 1 | 2 | 0 |
| 92 | C28H35NaO17 | 666.17719 | RPLC | 2.66 |  | 2.66 | 1 |  | 2 | 0 |


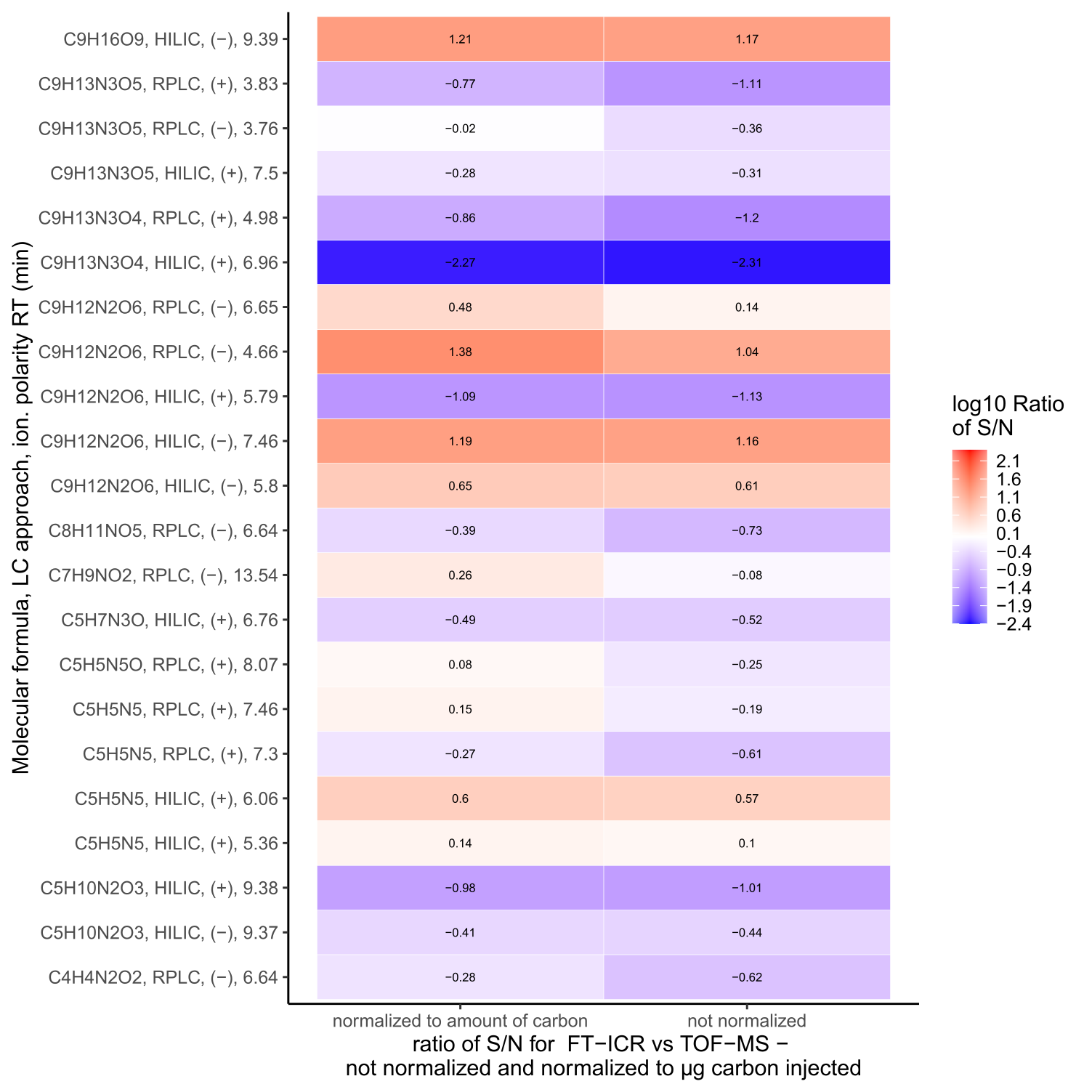


Figure S8: Heatmap for the comparison of the *S/N* ratio of matched, significant, *rth3*-mutant up-regulated compounds.

Only selected matches (molecular formula, LC approach, ionization polarity, retention time) are shown were both analytical approaches indicated up-regulation in the *rth3*-mutant for the same mass. Colours and numbers in the heatmap indicate the log_10_ of the *S/N* ratio of the same feature of soil column 12 for FT-ICR-MS/TOF-MS not normalized and normalized to the estimated amount of carbon injected for each feature for each of the analytical approaches. Negative values indicate a higher S/N for a compound with TOF-MS (blue), positive values a higher S/N with FT-ICR-MS (red).


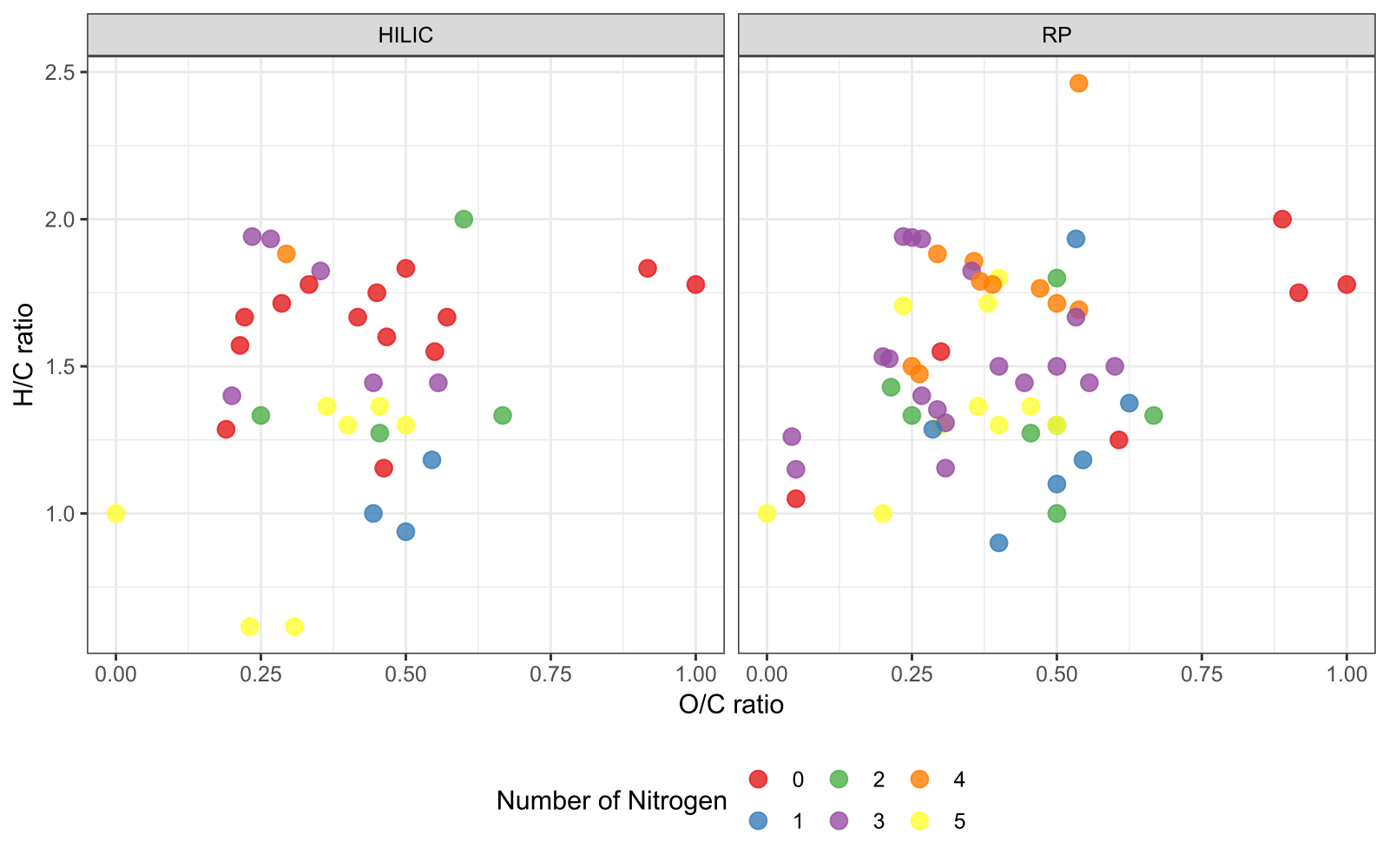


Figure S9: Van Krevelen diagram for matches of FT-ICR-MS with RPLC-TOF-MS or HILIC-TOF-MS.

Only matches are shown were both analytical approaches indicated up-regulation in the *rth3*-mutant for the same mass. The color indicates the number of nitrogen atoms in the molecular formula.

Table S7: Result of KEGG-query: putative *Zea mays* metabolites.

Significant, *rth3*-mutant up-regulated compounds matched HILIC/RPLC-TOF-MS with FT-ICR-MS after using the KEGG-pathway analysis [12]. Molecular formulas with an overlap of *Zea mays* metabolites are shown. 49 different metabolites were found, belonging to 33 pathways (132 combinations).

| **Pathway-ID** | **Pathway Description (number of metabolites)/ KEGG-ID’s belonging to the pathway** | **Metabolite Name** |
| --- | --- | --- |
| zma01100 | Metabolic pathways - Zea mays (maize) (39) |  |
|  | C00064 | L-Glutamine |
|  | C00089 | Sucrose |
|  | C00106 | Uracil |
|  | C00147 | Adenine |
|  | C00185 | Cellobiose |
|  | C00208 | Maltose |
|  | C00212 | Adenosine |
|  | C00214 | Thymidine |
|  | C00242 | Guanine |
|  | C00243 | Lactose |
|  | C00252 | Isomaltose |
|  | C00299 | Uridine |
|  | C00330 | Deoxyguanosine |
|  | C00387 | Guanosine |
|  | C00475 | Cytidine |
|  | C00559 | Deoxyadenosine |
|  | C00819 | D-Glutamine |
|  | C00822 | Dopaquinone |
|  | C00881 | Deoxycytidine |
|  | C01083 | alpha,alpha-Trehalose |
|  | C01235 | alpha-D-Galactosyl-(1->3)-1D-myo-inositol |
|  | C01252 | 4-(2-Aminophenyl)-2,4-dioxobutanoate |
|  | C02067 | Pseudouridine |
|  | C02376 | 5-Methylcytosine |
|  | C04785 | 13(S)-HPOT |
|  | C05402 | Melibiose |
|  | C05604 | 2-Carboxy-2,3-dihydro-5,6-dihydroxyindole |
|  | C05956 | Prostaglandin G2 |
|  | C05962 | 6-Keto-prostaglandin E1 |
|  | C05964 | 11-Dehydro-thromboxane B2 |
|  | C11544 | 2-O-(alpha-D-Mannosyl)-D-glycerate |
|  | C18054 | 2-Amino-2-deoxyisochorismate |
|  | C19631 | 6-Hydroxy-3-succinoylpyridine |
|  | C19972 | 2,4-Bis(acetamido)-2,4,6-trideoxy-beta-L-altropyranose |
|  | C20424 | 2,4-Diacetamido-2,4,6-trideoxy-D-mannopyranose |
|  | C20934 | 3-Deoxy-D-glycero-D-galacto-non-2-ulopyranosonate |
|  | C21029 | (R)-3-Ureidoisobutyrate |
|  | C22139 | gamma-L-Glutamyl-L-propargylglycine |
|  | C22254 | 2,4-Diacetamido-2,4,6-trideoxy-beta-L-gulose |
| zma02010 | ABC transporters - Zea mays (maize) (14) |  |
|  | C00064 | L-Glutamine |
|  | C00089 | Sucrose |
|  | C00185 | Cellobiose |
|  | C00208 | Maltose |
|  | C00212 | Adenosine |
|  | C00243 | Lactose |
|  | C00299 | Uridine |
|  | C00330 | Deoxyguanosine |
|  | C00387 | Guanosine |
|  | C00475 | Cytidine |
|  | C00559 | Deoxyadenosine |
|  | C00881 | Deoxycytidine |
|  | C01083 | alpha,alpha-Trehalose |
|  | C05402 | Melibiose |
| zma01110 | Biosynthesis of secondary metabolites - Zea mays (maize) (10) |  |
|  | C00089 | Sucrose |
|  | C00147 | Adenine |
|  | C00208 | Maltose |
|  | C01083 | alpha,alpha-Trehalose |
|  | C04785 | 13(S)-HPOT |
|  | C16321 | 9(S)-HPOT |
|  | C16341 | 2(R)-HPOT |
|  | C18054 | 2-Amino-2-deoxyisochorismate |
|  | C21923 | 9-Hydroxy-12-oxo-10(E),15(Z)-octadecadienoic acid |
|  | C22139 | gamma-L-Glutamyl-L-propargylglycine |
| zma00240 | Pyrimidine metabolism - Zea mays (maize) (9) |  |
|  | C00064 | L-Glutamine |
|  | C00106 | Uracil |
|  | C00214 | Thymidine |
|  | C00299 | Uridine |
|  | C00475 | Cytidine |
|  | C00881 | Deoxycytidine |
|  | C02067 | Pseudouridine |
|  | C02376 | 5-Methylcytosine |
|  | C21029 | (R)-3-Ureidoisobutyrate |
| zma00230 | Purine metabolism - Zea mays (maize) (7) |  |
|  | C00064 | L-Glutamine |
|  | C00147 | Adenine |
|  | C00212 | Adenosine |
|  | C00242 | Guanine |
|  | C00330 | Deoxyguanosine |
|  | C00387 | Guanosine |
|  | C00559 | Deoxyadenosine |
| zma00500 | Starch and sucrose metabolism - Zea mays (maize) (6) |  |
|  | C00089 | Sucrose |
|  | C00185 | Cellobiose |
|  | C00208 | Maltose |
|  | C00252 | Isomaltose |
|  | C01083 | alpha,alpha-Trehalose |
|  | C01725 | Levanbiose |
| zma00052 | Galactose metabolism - Zea mays (maize) (6) |  |
|  | C00089 | Sucrose |
|  | C00243 | Lactose |
|  | C01235 | alpha-D-Galactosyl-(1->3)-1D-myo-inositol |
|  | C05400 | Epimelibiose |
|  | C05401 | 3-beta-D-Galactosyl-sn-glycerol |
|  | C05402 | Melibiose |
| zma01250 | Biosynthesis of nucleotide sugars - Zea mays (maize) (4) |  |
|  | C19972 | 2,4-Bis(acetamido)-2,4,6-trideoxy-beta-L-altropyranose |
|  | C20424 | 2,4-Diacetamido-2,4,6-trideoxy-D-mannopyranose |
|  | C20934 | 3-Deoxy-D-glycero-D-galacto-non-2-ulopyranosonate |
|  | C22254 | 2,4-Diacetamido-2,4,6-trideoxy-beta-L-gulose |
| zma00592 | alpha-Linolenic acid metabolism - Zea mays (maize) (4) |  |
|  | C04785 | 13(S)-HPOT |
|  | C16321 | 9(S)-HPOT |
|  | C16341 | 2(R)-HPOT |
|  | C21923 | 9-Hydroxy-12-oxo-10(E),15(Z)-octadecadienoic acid |
| zma00590 | Arachidonic acid metabolism - Zea mays (maize) (3) |  |
|  | C05956 | Prostaglandin G2 |
|  | C05962 | 6-Keto-prostaglandin E1 |
|  | C05964 | 11-Dehydro-thromboxane B2 |
| zma00520 | Amino sugar and nucleotide sugar metabolism - Zea mays (maize) (3) |  |
|  | C19972 | 2,4-Bis(acetamido)-2,4,6-trideoxy-beta-L-altropyranose |
|  | C20424 | 2,4-Diacetamido-2,4,6-trideoxy-D-mannopyranose |
|  | C20934 | 3-Deoxy-D-glycero-D-galacto-non-2-ulopyranosonate |
| zma00380 | Tryptophan metabolism - Zea mays (maize) (2) |  |
|  | C01252 | 4-(2-Aminophenyl)-2,4-dioxobutanoate |
|  | C05835 | 2-Formaminobenzoylacetate |
| zma01240 | Biosynthesis of cofactors - Zea mays (maize) (2) |  |
|  | C00064 | L-Glutamine |
|  | C11355 | 4-Amino-4-deoxychorismate |
| zma00350 | Tyrosine metabolism - Zea mays (maize) (2) |  |
|  | C00822 | Dopaquinone |
|  | C05604 | 2-Carboxy-2,3-dihydro-5,6-dihydroxyindole |
| zma00470 | D-Amino acid metabolism - Zea mays (maize) (2) |  |
|  | C00064 | L-Glutamine |
|  | C00819 | D-Glutamine |
| zma00965 | Betalain biosynthesis - Zea mays (maize) (2) |  |
|  | C00822 | Dopaquinone |
|  | C05604 | 2-Carboxy-2,3-dihydro-5,6-dihydroxyindole |
| zma00460 | Cyanoamino acid metabolism - Zea mays (maize) (1) |  |
|  | C05711 | gamma-Glutamyl-beta-cyanoalanine |
| zma00630 | Glyoxylate and dicarboxylate metabolism - Zea mays (maize) (1) |  |
|  | C00064 | L-Glutamine |
| zma00790 | Folate biosynthesis - Zea mays (maize) (1) |  |
|  | C11355 | 4-Amino-4-deoxychorismate |
| zma00051 | Fructose and mannose metabolism - Zea mays (maize) (1) |  |
|  | C11544 | 2-O-(alpha-D-Mannosyl)-D-glycerate |
| zma00760 | Nicotinate and nicotinamide metabolism - Zea mays (maize) (1) |  |
|  | C19631 | 6-Hydroxy-3-succinoylpyridine |
| zma00750 | Vitamin B6 metabolism - Zea mays (maize) (1) |  |
|  | C00064 | L-Glutamine |
| zma00340 | Histidine metabolism - Zea mays (maize) (1) |  |
|  | C16673 | Isoglutamine |
| zma00970 | Aminoacyl-tRNA biosynthesis - Zea mays (maize) (1) |  |
|  | C00064 | L-Glutamine |
| zma00250 | Alanine, aspartate and glutamate metabolism - Zea mays (maize) (1) |  |
|  | C00064 | L-Glutamine |
| zma00220 | Arginine biosynthesis - Zea mays (maize) (1) |  |
|  | C00064 | L-Glutamine |
| zma00770 | Pantothenate and CoA biosynthesis - Zea mays (maize) (1) |  |
|  | C00106 | Uracil |
| zma00910 | Nitrogen metabolism - Zea mays (maize) (1) |  |
|  | C00064 | L-Glutamine |
| zma00410 | beta-Alanine metabolism - Zea mays (maize) (1) |  |
|  | C00106 | Uracil |
| zma00561 | Glycerolipid metabolism - Zea mays (maize) (1) |  |
|  | C05401 | 3-beta-D-Galactosyl-sn-glycerol |
| zma00908 | Zeatin biosynthesis - Zea mays (maize) (1) |  |
|  | C00147 | Adenine |
| zma01230 | Biosynthesis of amino acids - Zea mays (maize) (1) |  |
|  | C00064 | L-Glutamine |
| zma00997 | Biosynthesis of various secondary metabolites - part 3 - Zea mays (maize) (1) |  |
|  | C22139 | gamma-L-Glutamyl-L-propargylglycine |

Supplemental References

1. Vetterlein D, Lippold E, Schreiter S, Phalempin M, Fahrenkampf T, Hochholdinger F, Marcon C, Tarkka M, Oburger E, Ahmed M, Javaux M, Schlüter S. Experimental platforms for the investigation of spatiotemporal patterns in the rhizosphere-laboratory and field scale. J. Plant. Nutr. Soil Sci. 2021; https://doi.org/10.1002/jpln.202000079

2. Wen T-J, Schnable PS. Analyses of Mutants of Three Genes that Influence Root Hair Development in Zea mays (Gramineae) Suggest that Root Hairs are Dispensable. American Journal of Botany. 1994;81:833–842.

3. Godzien J, Alonso-Herranz V, Barbas C, Armitage EG. Controlling the quality of metabolomics data: new strategies to get the best out of the QC sample. Metabolomics. 2015; https://doi.org/10.1007/s11306-014-0712-4

4. Si-Hung L, Causon TJ, Hann S. Comparison of fully wettable RPLC stationary phases for LC-MS-based cellular metabolomics. Electrophoresis. 2017; https://doi.org/10.1002/elps.201700157

5. Da Silva MP, Kaesler JM, Reemtsma T, Lechtenfeld OJ. Absorption Mode Spectral Processing Improves Data Quality of Natural Organic Matter Analysis by Fourier-Transform Ion Cyclotron Resonance Mass Spectrometry. J. Am. Soc. Mass Spectrom. 2020; https://doi.org/10.1021/jasms.0c00138&ref=pdf

6. Lechtenfeld OJ, Kattner G, Flerus R, McCallister SL, Schmitt-Kopplin P, Koch BP. Molecular transformation and degradation of refractory dissolved organic matter in the Atlantic and Southern Ocean. Geochim. Cosmochim. Acta. 2014; https://doi.org/10.1016/j.gca.2013.11.009

7. Koch BP, Dittmar T, Witt M, Kattner G. Fundamentals of molecular formula assignment to ultrahigh resolution mass data of natural organic matter. Anal. Chem. 2007; https://doi.org/10.1021/ac061949s

8. Koch BP, Kattner G, Witt M, Passow U. Molecular insights into the microbial formation of marine dissolved organic matter: recalcitrant or labile? Biogeosciences. 2014; https://doi.org/10.5194/bg-11-4173-2014

9. Herzsprung P, Hertkorn N, Tümpling W von, Harir M, Friese K, Schmitt-Kopplin P. Understanding molecular formula assignment of Fourier transform ion cyclotron resonance mass spectrometry data of natural organic matter from a chemical point of view. Anal. Bioanal. Chem. 2014; https://doi.org/10.1007/s00216-014-8249-y

10. Kind T, Fiehn O. Seven Golden Rules for heuristic filtering of molecular formulas obtained by accurate mass spectrometry. BMC Bioinform. 2007; https://doi.org/10.1186/1471-2105-8-105

11. Herzsprung P, V Tümpling W, Hertkorn N, Harir M, Friese K, Schmitt-Kopplin P. High-field FTICR-MS data evaluation of natural organic matter: are CHON5S2 molecular class formulas assigned to (13)C isotopic m/z and in reality CHO components? Anal Chem. 2015; https://doi.org/10.1021/acs.analchem.5b02549

12. Kanehisa M, Sato Y, Kawashima M. KEGG mapping tools for uncovering hidden features in biological data. Protein Sci. 2022; https://doi.org/10.1002/pro.4172
